# What lies beneath: *Hydra* provides cnidarian perspectives into the evolution of FGFR docking proteins

**DOI:** 10.1007/s00427-020-00659-4

**Published:** 2020-03-20

**Authors:** Ashwini Suryawanshi, Karolin Schaefer, Oliver Holz, David Apel, Ellen Lange, David C. Hayward, David J. Miller, Monika Hassel

**Affiliations:** 1 Morphology and Evolution of Invertebrates, Philipps University, FB17, Karl von Frisch Str. 8, 35032 Marburg, Germany; 2 DFG Research Training Group, Membrane Plasticity in Tissue Development and Remodeling, GRK 2213, Philipps-Universität Marburg, Marburg, Germany; 3 Research School of Biology, Australian National University, Canberra, ACT 0200 Australia; 4 ARC Centre of Excellence for Coral Reef Studies, James Cook University, Townsville, Queensland 4811 Australia

**Keywords:** Receptor tyrosine kinase, Adapter protein, Grb2, Crkl, Dof

## Abstract

Across the Bilateria, FGF/FGFR signaling is critical for normal development, and in both *Drosophila* and vertebrates, docking proteins are required to connect activated FGFRs with downstream pathways. While vertebrates use Frs2 to dock FGFR to the RAS/MAPK or PI3K pathways, the unrelated protein, downstream of FGFR (Dof/stumps/heartbroken), fulfills the corresponding function in *Drosophila*. To better understand the evolution of the signaling pathway downstream of FGFR, the available sequence databases were screened to identify Frs2, Dof, and other key pathway components in phyla that diverged early in animal evolution. While Frs2 homologues were detected only in members of the Bilateria, canonical Dof sequences (containing Dof, ankyrin, and SH2/SH3 domains) were present in cnidarians as well as bilaterians (but not in other animals or holozoans), correlating with the appearance of FGFR. Although these data suggested that Dof coupling might be ancestral, gene expression analysis in the cnidarian *Hydra* revealed that *Dof* is not upregulated in the zone of strong *FGFRa* and *FGFRb* expression at the bud base, where FGFR signaling controls detachment. In contrast, transcripts encoding other, known elements of FGFR signaling in Bilateria, namely the FGFR adaptors Grb2 and Crkl, which are acting downstream of Dof (and Frs2), as well as the guanyl nucleotide exchange factor Sos, and the tyrosine phosphatase Csw/Shp2, were strongly upregulated at the bud base. Our expression analysis, thus, identified transcriptional upregulation of known elements of FGFR signaling at the *Hydra* bud base indicating a highly conserved toolkit. Lack of transcriptional *Dof* upregulation raises the interesting question, whether *Hydra* FGFR signaling requires either of the docking proteins known from Bilateria.

**Electronic supplementary material:**

The online version of this article (10.1007/s00427-020-00659-4) contains supplementary material, which is available to authorized users.

## Introduction

Across the Bilateria, fibroblast growth factor receptors (FGFR) and their ligands control embryonic as well as adult morphogenesis. Although the receptor tyrosine kinase (RTK) superfamily has earlier origins, the FGF/FGFR signaling system is thought to have evolved in Ureumetazoa, the last common ancestor of Cnidaria and Bilateria (Babonis and Martindale 2017; Bertrand et al. 2014; Lange et al. 2014; Oulion et al. 2012; Rebscher et al. 2009). Little is known about the evolution of signaling elements downstream of this specific family of receptor tyrosine kinases. In both the fly and vertebrates, docking proteins are essential to specifically transduce the signal of an activated (trans-phosphorylated) FGFR dimer into the cell (Brummer et al. 2010; Lemmon and Schlessinger 2010).

One specific issue of interest is the nature of the ancestral mechanism by which the activated FGFR is coupled to downstream signaling pathways, as vertebrates, *Drosophila* and the nematode *C. elegans* use completely unrelated proteins to fulfill this task. In vertebrates, Frs2 (FGF receptor **s**ubstrate **2**), a member of the membrane-linked protein (MLP) family, connects FGFR to the PI3 kinase and RAS/ERK1/2 signaling pathways (Gotoh 2008). In *Drosophila,* Dof (downstream **o**f FGFR, also known as stumps or heartbroken), is essential for FGFR signaling and connects the heartless and breathless FGFRs to the RAS/MAPK or PI3 kinase signaling pathways (Csiszar et al. 2010; Michelson et al. 1998; Muha and Muller 2013; Vincent et al. 1998). In both cases, the activated FGFR dimer phosphorylates conserved tyrosines in the docking proteins and generates secondary binding sites for the intracellular adapters Grb2 (Kouhara et al. 1997), Crk and Crkl (Birge et al. 2009) as well as the tyrosine phosphatase Shp2/Csw (syn. Corkscrew, Csw, in *Drosophila*) (Gotoh 2008; Hadari et al. 1998; Lax et al. 2002) and the dual specificity guanine nucleotide exchange factor (GEF) Sos, that regulates both Ras and Rac family GTPases (Innocenti et al. 2002). In the nematode *C. elegans*, Grb2 is the only known FGFR docking protein: Frs2 has no FGFR docking function and the genome does not encode a Dof homologue (Lo et al. 2010).

Grb2 is an interesting protein, because it may act as an adapter as well as a docking protein downstream of vertebrate and invertebrate FGFRs. It has an intrinsic FGFR binding activity in both the phosphorylated and unphosphorylated forms and exerts multiple functions on FGFR. In vertebrates, unphosphorylated Grb2 is associated constitutively to the C-terminal domain of inactive FGFR2 dimers, preventing unwanted activation (Belov and Mohammadi 2012; Lin et al. 2012). Upon receptor activation, Grb2 dissociates from such FGFR pairs and only then serves as adaptor between Frs2 and Sos or Shp2.

The FGFR docking proteins Frs2 or Dof, the adapters Grb2, Crk, and Crkl, the GEF Sos and Shp2 thus constitute, in various combinations, an essential toolkit in vertebrate, fly, and worm to control FGF-induced signal transduction in, e.g., cell migration or neuronal differentiation (Bottcher and Niehrs 2005; Muha and Muller 2013; Zhou et al. 2015).

Since Dof and Frs2 dock FGFR in a mutually exclusive manner in fly and vertebrate respectively, and neither are required for FGFR signaling in the nematode, the phylogenetic distributions of docking and downstream signaling components were surveyed, focusing particularly on FGFR docking proteins. Included in this survey were representatives of early diverging animal phyla. Among these, the Cnidaria are of most interest, because FGF signaling is thought to have its origins in the eumetazoan common ancestor (Bertrand et al. 2014), and thus prior to the Cnidaria/Bilateria divergence, which occurred at or near the Ediacaran/Cambrian boundary (Schwaiger et al. 2014).

FGFR signaling has been shown to be essential for development in two evolutionarily distant cnidarians. In *Nematostella vectensis* (Anthozoa) larvae, FGFR/RAS/MAPK signaling is required for the development of the apical organ, a sensory ciliated tuft (Matus et al. 2007; Rentzsch et al. 2008). In the freshwater polyp *Hydra*, FGFR signaling is indispensable for at least two steps of the vegetative budding process. While the FGFR/MEK/dpERK pathway modulates timing of *Hydra* bud detachment (Hasse et al. 2014; Sudhop et al. 2004), an FGFR/Rho/Rock/myosin II pathway controls cell shape changes required for constriction and separation of the tissue bridge connecting parent and bud (Holz et al. 2017).

In the present study, the available sequence databases were scanned for non-bilaterian homologues of Dof/stumps/heartbroken, Frs2, and other key downstream components of the FGFR pathway. Whereas likely Dof orthologues were detected in several cnidarians, canonical Frs2 sequences could not be identified in any non-bilaterians, suggesting that the ancestral (invertebrate) FGFR was Dof-coupled. To test this hypothesis, in situ hybridization was used to investigate the expression pattern of Dof in *Hydra* in relation to those of the FGFRs. Surprisingly, Dof transcripts were not detected in zones of strong FGFR gene expression. In contrast to *Dof*, the transcripts encoding downstream components *Grb2*, *Crkl*, *Sos*, and *Shp2* were strongly and specifically upregulated at the bud base together with both of the *Hydra FGFRs.* Presence of a highly conserved toolkit for FGFR downstream signaling is thus indicated, but whether Dof functions in *Hydra* FGFR signaling needs future investigation.

## Materials and methods

### Gene prediction

To reveal Frs2 and Dof sequences in *Hydra*, we explored the NCBI (http://www.ncbi.nlm.nih.gov), JGI (http://jgi.doe.gov/), hydrazome/metazome (http://hydrazome.metazome.net/cgi-bin/gbrowse/hydra), Compagen (http://www.compagen.org) T-CDS: transcript models (contigs) derived from assembled ESTs (Hemmrich and Bosch 2008) and RNASeq project (Wenger and Galliot 2013). Predicted protein sequences were further analyzed for conserved domains using NCBI’s conserved domain search tool including CDART (http://www.ncbi.nlm.nih.gov/Structure/cdd/wrpsb.cgi) (Geer et al. 2002; Marchler-Bauer et al. 2015), ExpasyProsite (http://prosite.expasy.org/), Pfam (http://pfam.sanger.ac.uk/), or PhosphoMotif finder (Amanchy et al. 2007). Motif Scan (Pagni et al. 2007) was used to predict domains and identify SH2, SH3-binding site consensus sequences in Dof sequences (http://scansite.mit.edu/cgi-bin/motifscan_seq, 28 July 2016). GPS 5.0 (http://gps.biocuckoo.cn/) was used to identify predicted phosphorylation sites for Crkl. BLAST search revealed homologous or related proteins by sequence similarity (BLAST search parameter: All non-redundant GenBank CDS translations + PDB + SwissProt + PIR + PRF excluding environmental samples from WGS projects). Figures depicting protein domains were established using the IBS illustrator (Liu et al. 2015).

### Phylogeny

Predicted *Hydra* protein sequences were aligned with the available protein sequences of the choanoflagellate *Salpingoeca*, the parazoan *Trichoplax*, the ctenophore *Mnemiopsis*, the Cnidaria *Acropora*, and *Nematostella* as well as protein sequences of several bilaterian animals covering protostome and deuterostome phyla as indicated in the figures. Alignments were calculated using ProbCons version 1.12 (Do et al. 2005), clustalX, T-coffee version 8.99 (Notredame et al. 2000), MAFFT L-INS-i version 7.037b (Katoh and Standley 2013), and the COBALT program (Papadopoulos and Agarwala 2007) with default settings. Jalview version 2.8 (Waterhouse et al. 2009) and InterProScan5 was used to visualize and analyze the alignments whereas Genedoc was used to manually edit them. Phylogenetic trees were calculated as indicated in the text using either conserved domains or the whole protein sequences and rooted as specified in the text. Gaps between sequences were deleted. The WAG + G + I model was selected as the best fitting amino acid substitution model according to the Bayesian information criterion in ProtTest version 3.3 (Darriba et al. 2011). Phylogenetic trees were calculated using Mr. Bayes 3.1.2 (Huelsenbeck and Ronquist 2001). Two runs were initiated of four Markov chain Monte Carlo (MCMC) chains of 2 × 10^7^generations, each from a random starting tree. Sampling made every 1000 generations [additional settings: rates = invgamma, ngammacat = 4, aamodelpr = WAG]. A 25% burn-in was selected and convergence was assessed by standard deviation of split frequencies falling below 0.005. The resultant trees were visualized with Figtree version 1.4.0 (http://tree.bio.ed.ac.uk/software/figtree/).

### *Hydra* culture

*Hydra vulgaris AEP* strain was cultured in a medium containing (0.29 mM CaCl_2_, 0.59 mM MgSO_4_, 0.5 mM NaHCO_3,_ and 0.08 mM K_2_CO_3_, pH 7.4) at 18 °C. The animals were fed 5 times a week with freshly hatched *Artemia nauplii* to synchronize their growth (Sudhop et al. 2004).

### Cloning of sequences

The Quickprep Micro Kit (Amersham) was used to harvest poly(A)^+^ RNA from the *Hydra vulgaris AEP* strain (*Dof, Frs2-related, Grb2*) or the *Hydra vulgaris Zürich* strain (Sos, Csw). Further poly(A)^+^ RNA was reverse transcribed using Revert Aid TM Premium First-strand cDNA Synthesis Kit (Fermentas) and diluted 1:100 prior to PCR amplification of the genes of interest. *Dof, Frs2*, *Sos*, *Shp2/Csw*, *Crkl*, *and Grb2* gene sequences were PCR amplified using following the primer pairs:

- *Dof* forward GTTGCAGTTTTTAATTCAAATATACC (111–137)
- *Dof* reverse TTGCAGCTGCTATGTCCATTGG (682–660)
- *Frs2-related* forward ATGGAGGTAATTTTGGAAGGC (1–21 bp)
- *Frs2-related* reverse: GACCTACTACATTCAAATCGA (566–545)
- Sos forward GGTTGATCTCCAAATGCACGA (-13–5)
- Sos reverse CGACGCTTAGCTAGTGGCTG (560–540)
- Shp2/Csw forward CGGCGTTTTTATTGAGCTGC (572–592)
- Shp2/Csw reverse CGAACACAGAGAGCTGGCAT (1463–1443)
- Grb2 forward CGCAGATCTGAGGCTGAACA (201–221)
- Grb2 reverse CGGTATTTTAGGAAGGGGGAGT (1090–1068)
- Crkl forward TCGGGTTACTGAGCCAACAC (294–1040)
- Crkl reverse CCAGGCGCTACATTAAAGGC (1021–1040)

The full length *Fgfr-b* cDNA was reconstituted by using two sequence fragments encoding the first two Ig-like loops (*fgfr-b*_*ex*_, 835 bp, and a fragment encoding the tyrosine kinase domain (*fgfr-b*_*i*n_, 516 bp), which had been identified previously in the *Hydra AEP* database (Hemmrich and Bosch 2008; Rudolf et al. 2013). The missing sequence between these two fragments was isolated from cDNA by PCR using proofreading polymerase (Pfu) and the following primers:

*FGFRb* forward CGTTTACAGCATGACAAATCC, *FGFRb* reverse CAAATGACCATATATCACTTCGAG. Accession numbers of the two previously existing fragments are HAEP_T-CDS_v02_12177 (Compagen database, encoding Ig-like loops I and II, named here: *FGFRb_ex*) and HAEP_T-CDS_v02_12974 (Compagen database, encodes the tyrosine kinase domain, named here: *FGFRb_in*). Amplified cDNA fragments were AT-cloned (*Dof*, *Frs2-related*, *Sos, Shp2/Csw*, *Grb2*, *Crkl*) into the pGEM T-Easy vector (Promega) or blunt end (*FGFRb*) into the CloneJET vector (ThermoFisher). Clone identity was confirmed by sequencing (SeqLab).

### Whole mount in situ hybridization

Full length (*FGFRa*, *Dof, Frs2-related, Sos, Shp2/Csw, Grb2, Crkl*) or partial sequences (*FGFRb*) were used for the synthesis of Dig-labeled RNA sense and antisense probes (ROCHE). *HAEP_Dof* RNA probe (571 bp: nucleotides 1496 to 2067); *HAEP_Frs2-related* RNA probe (561 bp: nucleotides 166 to 727); *Hvz_Sos* RNA probe (889 bp); *Hvz_Shp2/Csw* RNA probe (1,191 bp: nucleotides 471 to 1,662); *Hvz_Grb2* RNA probe (889 bp: nucleotides 674 to 1,563); *FGFRb*_*in*_, 516 bp and *FGFRb*_*ex*_, 835 bp (two probes were necessary to detect and exclude a cross reaction of the two probes with parts of the *FGFRa* mRNA encoding the highly conserved tyrosine kinase domain). Whole mount in situ hybridization was performed as described previously (Sudhop et al. 2004) with the exception that proteinase K digestion was prolonged for *Hydra vulgaris AEP* from 10 to 15 min. Bud stages were selected according to (Otto and Campbell 1977). The quality of RNA probes was verified by Northern blotting and between 3 and 300 ng of the respective RNA probe were used for WMISH to obtain an optimal signal-to-noise ratio. For each in situ hybridization, at least 5 polyps of a given bud stage were used and the expression pattern is described only if at least 4 of those (80%) show the same pattern in independent experiments. Nonspecific binding patterns of probe and/or antibody which is are unrelated to specific probes are given as examples in Fig. ESM8.

## Results

### Frs2 and Frs2-related proteins as FGFR adaptors

**Fig. 1:**
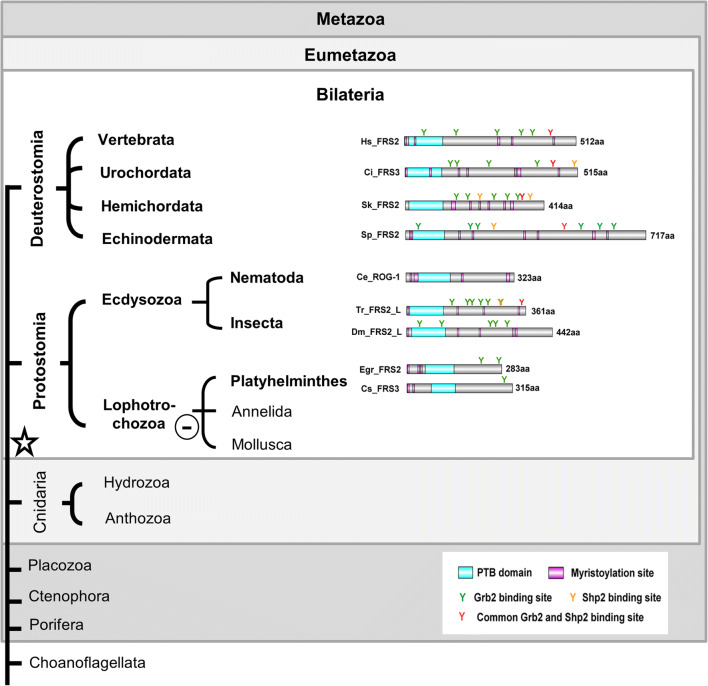
Schematic summary of structural features of metazoan Frs2 homologues. FRS2 homologues were identified in Bilateria only. Ce_ ROG1, *Caenorhabditis elegans*; Ci_ FRS3, *Ciona intestinalis*; Cs_FRS3, *Clonorchis sinensis*; Dm_FRS2, *Drosophila melanogaster;* Egr_FRS2, *Echinococcus granulosus*; Hs_FRS2*, Homo sapiens*; Sk_ FRS2, *Saccoglossus kowalevskii*; Sp_ FRS2, *Strongylocentrotus purpuratus;* Tr_ FRS2, *Tribolium castaneum*

The FGFR docking proteins of vertebrates, Frs2 homologues, typically, carry an N-terminal myristoylation site (Fig. 1), which ensures their modification by a lipid anchor and constitutive localization to the plasma membrane. A phosphotyrosine-binding (PTB) domain (pfam08416) links Frs2 constitutively to activated vertebrate FGF receptors, and multiple tyrosine phosphorylation sites are essential to dock downstream adaptor proteins like Grb2 or the phosphatase Shp2/Csw via SH2 and SH3 domain binding consensus sequences (Brummer et al. 2010; Gotoh 2009).

Querying the sequence databases with the known Frs2 proteins yielded convincing matches only for deuterostomes, ecdysozoans, and flat worms (Fig. 1). Only in proteins from these groups could the presence of an N-terminal myristoylation sequence (as well as of a PTB domain) be confirmed. In representatives of the Mollusca, Annelida, and Cnidaria, “Frs2-related” proteins were identified (Fig. ESM1A, Fig. ESM1B). Although in these cases a conserved PTB domain sequence was present, a PH (pleckstrin homology) domain replaced the diagnostic N-terminal myristoylation site at the N-terminus. This domain (PH/PTB) combination is characteristic of Dok and IRS proteins, which together with Frs2 form the membrane-linked protein (MLP) superfamily (ESM2A-C). PH domains bind phospholipids and anchor proteins to membranes in an analogous manner to the myristoyl tail (Delahaye et al. 2000; Uhlik et al. 2005). The “Frs2-related” proteins identified here in annelids, mollusk, and cnidarians are clearly members of the MLP superfamily (ESM2), but not of the Frs2 sensu *strictu* clade.

A major challenge in uncovering relationships between the invertebrate/non-metazoan Frs2-related sequences recovered and the true Frs2 proteins of vertebrates and insects was the low sequence similarity, and for many of the former it is difficult to make firm assignments. The choanoflagellate matches, including the *Salpingoeca* sequence XP_004995975, are unconvincing—this latter sequence has a cyclophilin type peptidylproply *cis*-trans isomerase domain as well as PH-like domain. The sponge sequence recovered as XP_003383468.1 is a homologue of proline-rich receptor-like protein kinase, PERK7. It lacks a PH domain, but does have a PTB domain. Although the *Nematostella* sequences XP_001635403.1 and XP_001641972.1 were the best hits in Frs2 BLAST searches, domain searches give stronger hits to the IRS type domain (pfam02174, IRS, PTB domain (IRS-1 type)), and the same is true for the *Acropora* database match. As for *Hydra*, there is, additional to the Frs2-related proteins, an IRS1-like protein annotated (Acc. No. XP_012558666.1), which lacks, however, the IR-binding domain (ESM2A, B). The presence of an N-terminal PH domain in the Frs2-related sequences from Placozoa, cnidarians, annelids, and mollusks rendered them similar to IRS or Dok proteins rather than Frs2. A phylogenetic analysis of Frs2 and Frs2-related protein sequences revealed no convincing relationships (not shown).

In summary, our data imply that true Frs2 proteins are likely to have evolved in Urbilateria and were lost again during the evolution of the lophotrochozoan phyla Annelida and Mollusca.

### Dof is a candidate FGFR docking protein in Cnidaria

**Fig. 2:**
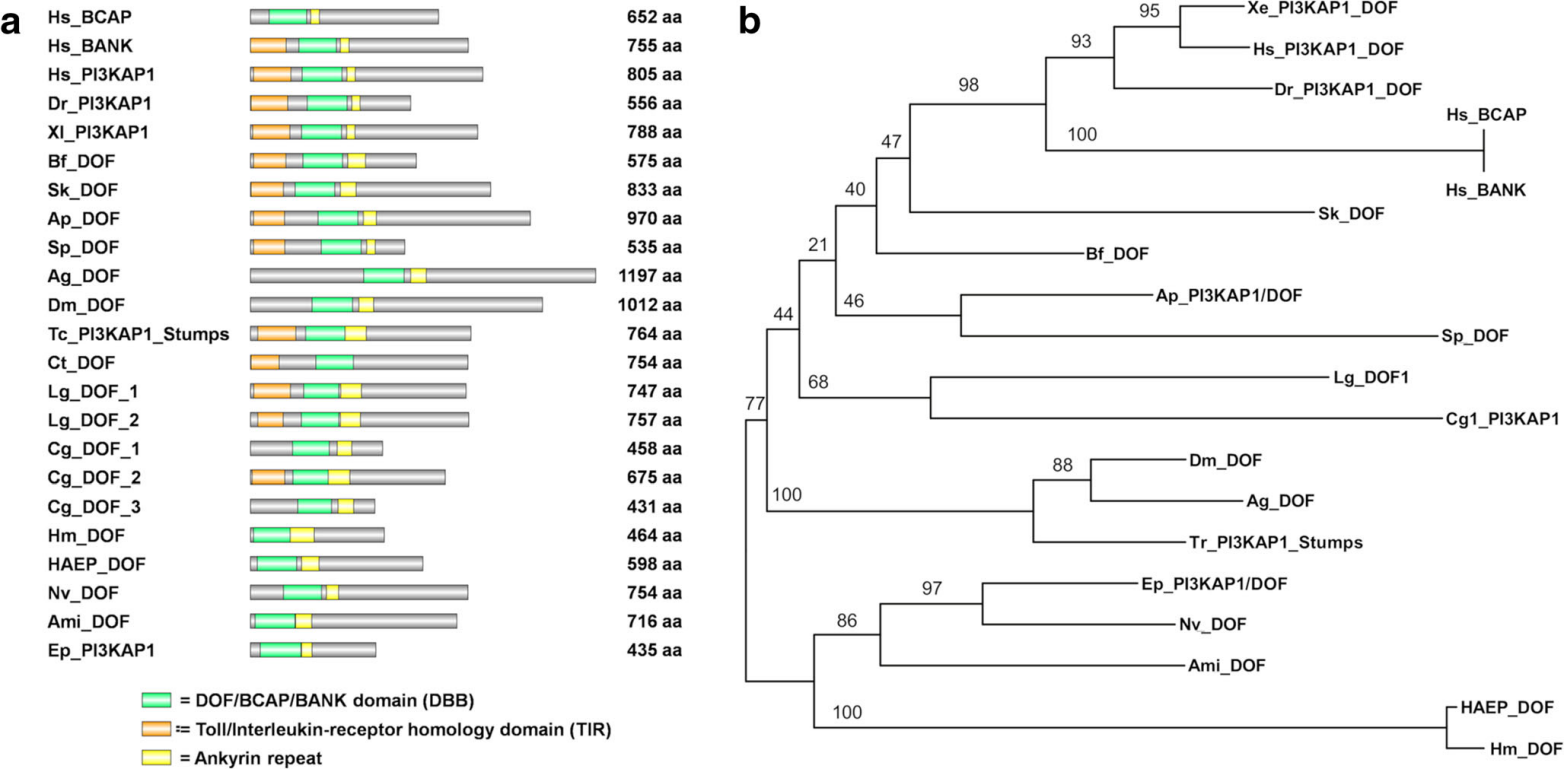
Structural features and phylogenetic relationship of eumetazoan Dof/PI3KAP proteins. (A) Protein structure of Dof/PI3KAP proteins. (B) Phylogenetic tree of Dof/PI3KAP proteins. Numbers at nodes indicate posterior probability support values. Ami, *Acropora millepora*; *Ag, Anopheles gambiae*; Ap, *Asterina*; Bf, *Branchiostoma floridae*; Cg, *Crassostrea gigas;* Ct, *Capitella teleta;* Dm, *Drosophila melanogaster;* Dr., *Danio rerio*; Ep, *Exaiptasia pallida*; HAEP, *Hydra vulgaris AEP*; Hm, *Hydra magnipapillata*; Hs, *Homo sapiens*; Lg, *Lottia gigantea*; Sk, *Saccoglossus kowalevskii; Sp, Strongylocentrotus purpuratus;* Tc, *Tribolium castaneum*; Xl, *Xenopus laevis*

All Dof proteins are characterized by the presence of the DBB (Dof, BCAP, and BANK) domain (pfam14545), which is required for binding to an activated receptor (Fig. 2A). They typically also contain a number of ankyrin repeats (Gotoh 2009; Muha and Muller 2013) which mediate protein–protein interactions in *Drosophila* (Battersby et al. 2003; Vincent et al. 1998; Wilson et al. 2004). Ankyrin repeats have also been maintained in the Dof paralogues BANK and BCAP. More recently annotated proteins were named PI3 kinase adapter proteins (PI3KAP), instead of Dof, due to their similarity to human BCAP, which interacts with PI3 kinase (Lauenstein et al. 2019).

Screening of the available genomic and EST databases with the sequences encoding the DBB domain and ankyrin repeats of fly Dof revealed ESTs encoding full length Dof homologues from *Hydra magnipapillata* and *Hydra vulgaris AEP* (Fig. 2A, Fig. ESM1A, ESM3). Although these differed significantly in size (*H. magnipapillata* Dof is predicted to be 464 amino acid (aa) residues, whereas that from *H. vulgaris AEP* is 598 aa), in terms of domain structure these are both typical Dof proteins. Similar search strategies applied to the data available for anthozoan cnidarians, led to the identification of *Dof* sequences in the corals *Acropora millepora, A. digitifera, Exaiptasia pallida*, and the sea anemone *Nematostella vectensis*. Alignment of these assembled Dof domain sequences with predicted or annotated Dofs from a range of bilaterians (ESM3) implies that these are likely to be orthologues. In each case, structure prediction programs (conserved domain search tool, NCBI) identified a DBB domain associated with an ankyrin repeat domain and multiple tyrosine phosphorylation consensus sequences for SH2- and SH3-binding domains of intracellular proteins such as Grb2, Crk, Shp2, PI3K, Src, or RasGAP, respectively (ESM4). Conspicuous was the presence of a structurally defined N-terminal Toll/Interleukin-receptor homology (TIR) domain in most of the bilaterian sequences and its lack in Cnidaria (Fig. 2A).

Although the presence of *Dof* genes in anthozoan and hydrozoan cnidarians as well as in a wide range of bilaterians implies that Dof may have mediated FGFR signaling in Ureumetazoa, homologues of this protein could not be identified in the parasitic flat worms (Platyhelminthes) *Clonorchis sinensis* and *Echinococcus granulosus*, in the nematode *C. elegans* or in the urochordate *Ciona intestinalis*. In some cases, failure to identify Dof homologues may reflect the quality of genome assembly/gene predictions, but in others (e.g., *C. elegans*) gene loss or high levels of sequence divergence are more likely explanations. Despite identification of likely Dof orthologues in several cnidarians, neither Dof nor its vertebrate paralogues BANK or BCAP could be identified in the ctenophore *Mnemiopsis*, the sponge *Amphimedon*, or in the placozoan *Trichoplax*. A phylogenetic analysis confirmed the homology of Dof proteins across the Eumetazoa (Fig. 2B).

### Candidate downstream elements of *Hydra FGFR: Grb2, Crkl, Sos*, and *Shp2/Csw*

As outlined above, the FGFR signal is transduced by coupling to several different intracellular signaling pathways in the fly and vertebrates, one of which—the RAS/MAPK pathway leading to Erk1/2 activation—is known to act downstream of FGFRs in both *Nematostella* and *Hydra* (Hasse et al. 2014; Matus et al. 2007; Rentzsch et al. 2008).

To enable further investigation of the signaling system downstream of *Hydra* FGFR, the cDNA sequences of *Grb2, Crkl, Sos*, and *Shp2/Csw* were retrieved from the databases (ESM5). These four proteins clearly all have early origins; the *Hydra* homologues of each closely resemble their bilaterian counterparts in terms of domain structures (ESM6, ESM7). Despite apparent anomalies with respect to some of the *Nematostella* data in GenBank (e.g., the *Nematostella* Grb2 protein entry features an N-terminally truncated SH2 domain (ESM6C)), all four components were also identified in the sponge *Amphimedon* (ESM5**)**. Homologues of Shp2 and Grb2 were also identified in the choanoflagellate *Salpingoeca* and *Monosiga*, so at least these two components pre-date metazoan origins.

In the case of *Drosophila*, database entry NP_651908.1 appears to have been misannotated as a homologue of Crk rather than of Crkl; Crk is a paralogue of Crkl that is only otherwise known in vertebrates (where both proteins are present). To clarify both the identity of NP_651908.1 and the evolutionary history of these proteins, phylogenetic analyzes of the Crk and Crkl proteins were undertaken. The resulting phylogenetic tree (ESM7B) indicates (i) that *Drosophila* NP_651908.1 falls in a well-supported clade with the nematode and mollusk Crkl homologues, and is well-resolved from true Crk homologues (implying that the database accession information may require modification) and (ii) that Crk likely resulted from a vertebrate-specific duplication of Crkl (ESM7 A, B).

In summary, a suite of proteins that are known to function downstream of FGFR across the Bilateria are also present in non-bilaterian animals and, although FGFR signaling evolved in the ureumetazoan common ancestor, several of the downstream components have earlier origins.

### Transcripts of FGFR downstream adaptors and effectors, but not Dof or Frs2-related, are upregulated together with the FGFRs at the bud detachment site

**Fig. 3:**
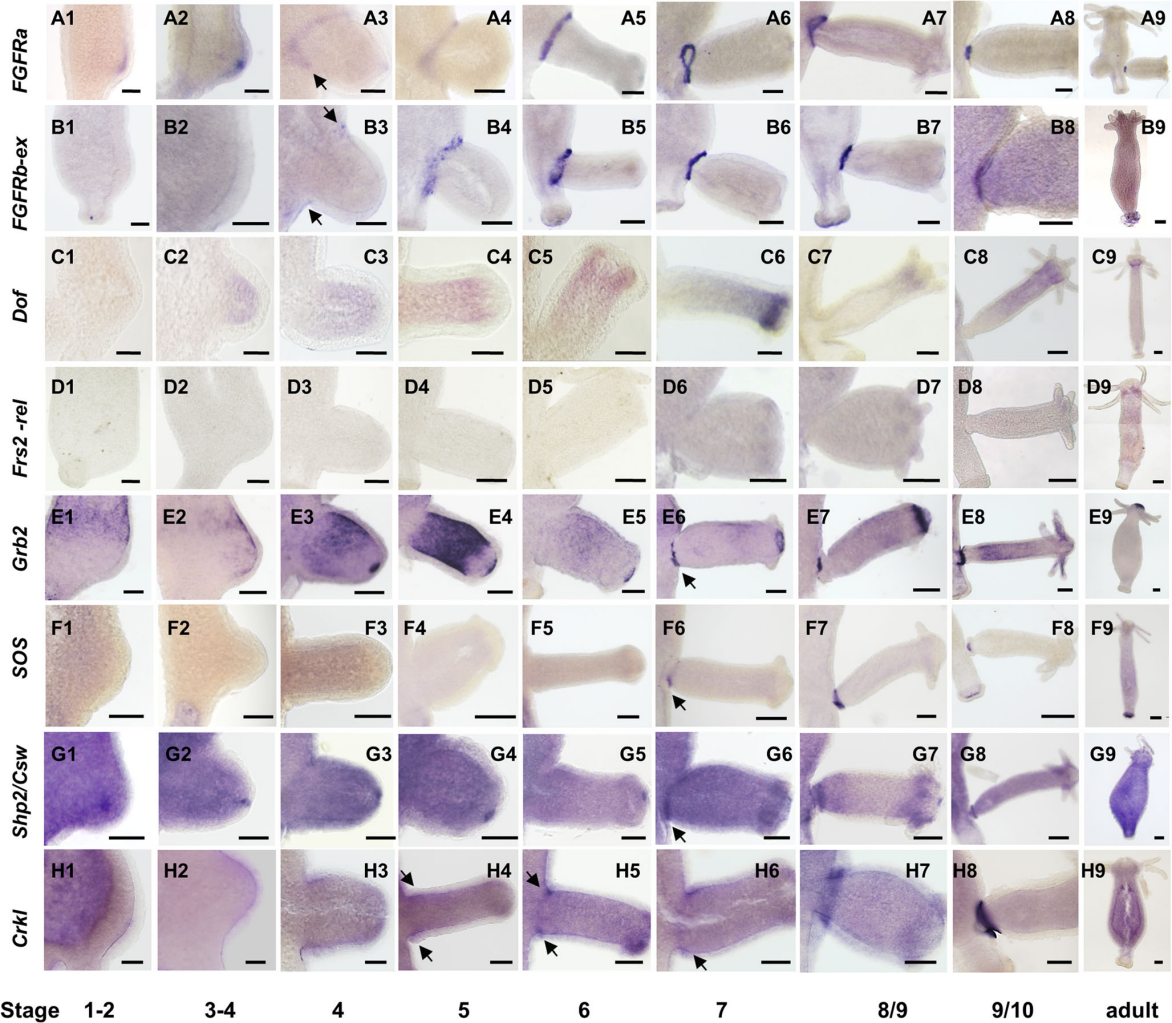
Expression patterns of *Hydra* FGFRs and their potential downstream signaling elements in the ten bud stages (according to Otto and Campbell 1977)

In order to function in FGFR signal transmission, pathway components must co-localize, and may therefore be spatio-temporally co-expressed. *Hydra* FGFRa (Kringelchen) transcripts have previously been shown to be upregulated at the bud base, but the gene is also expressed weakly throughout the body column of *Hydra vulgaris Zurich* and *Hydra vulgaris Ind-Pune* (Sudhop et al. 2004; Turwankar and Ghaskadbi 2019). FGFR signaling is indispensable for bud detachment (Hasse et al. 2014; Holz et al. 2017), and therefore, the expression domains of the *Hydra Dof*, *Frs2-related, Grb2, Crkl, Sos*, and *Shp2/Csw* homologues were compared to those of the *Hydra vulgaris* FGFRs (*FGFRa* and *FGFRb)* in the budding process. Since the *Hydra* FGFRs diverge only in their N-terminal regions, their expression was detected using probes corresponding to either the full length FGFRa or to the divergent N-terminal extracellular domain of FGFRb. Both *FGFR* transcripts as well as those of *Dof, Frs2-related*, and *Sos* were found weakly (and constitutively) expressed along the non-budding body column (Fig. 3(A)–(D) and (F); sense controls in ESM8). *Grb2, Shp2*, and *Crkl*, in contrast, were expressed strongly and their specific expression patterns detectable only by using highly diluted probes (Fig. 3E, G, H; sense controls in ESM8). As reported previously (Sudhop et al. 2004), *kringelchen* (FGFRa) expression is upregulated from stage 2 to stage 4 at the bud tip and from stage 4 onwards at the bud base. The *FGFRb(ex)* probe failed to detect early expression in the bud tip, but colocalized with *FGFRa* at the bud base from stage 4 (Fig. 3(B4)–(B8)). Of the docking protein transcripts, only *Dof* was found co-expressed with *FGFRa* in bud evagination stages 3 and 4 (Fig. 3(C2)–(C3)). Neither *Dof* nor *Frs2-related* transcripts colocalized in the strong *FGFRa* and *FGFRb* expression domains at the bud base*. Dof* mRNA was localized in the upper body region of developing buds as well as later, constitutively in the tentacle zone (Fig. 3(C5)–(C9)), while expression of *Frs2-*related was only detected at low levels at the tentacle bases (Fig. 3(D6)–(D9)).

In the bud detachment phase, both FGFR transcripts are strongly co-expressed with *Grb2, Sos, Shp2/Csw*, and *Crkl* at the bud base from stages 6 to 7 onwards (Fig. 3e, f, g, h), low levels of Crkl expression being already observed in stage 5 (Fig. 3(H4)) and strongly from stage 8 onwards (Fig. 3(H7)–(H8)). Of the downstream components tested, *Crkl* was the first to be upregulated at the bud base.

In addition to expression in the bud detachment zone, and by implication therefore not related to signaling via FGFRs, *Grb2* and *Shp2/Csw* displayed complex expression patterns in the *Hydra* endoderm, and both ecto- and endoderm, respectively. *Shp2/Csw* transcription was upregulated at the bud tip in both epithelia from stages 1 to 2 onwards and persisted until shortly before the bud detached in stage 10 (Fig. 3(G1)–(G7)). No transcripts were detectable in the adult head.

*Grb2* transcription was dynamic in budding polyps (Fig. 3(E1)–(E9); ESM8 I, J). Superimposed on ubiquitous background expression, *Grb2* was upregulated endodermally in a circumferential belt of parental tissue immediately above the newly forming bud and persisted in a wedged expression zone until stage 3/4 (Fig. 3(E1); ESM8 I). In tissue transferred to the bud (Fig. 3(E3)–(E5)), expression intensity increased in bud stages 4–5. From stage 4 onwards, *Grb2* was also highly expressed in the bud tip, where the mouth opening developed (Fig. 3(E3)–(E8)). Expression in this region persisted in the adult in a ring of cells surrounding the mouth (Fig. 3(E9)). The intensity of this apical staining was variable as documented in Fig. ESM8 J, compare bud to parent). From stage 7 onwards, expression in the body column of the bud ceased and a strong signal developed in cells surrounding the bud base, trailing behind Crkl (Fig. 3(E6)–(E8)). The dynamic pattern of *Grb2* expression suggests that the corresponding protein fulfills multiple roles during bud development in *Hydra*.

Taken together, the *FGFRs, Grb2, Crkl, Sos,* and *Shp2/Csw* are all expressed at the late bud base and might thus form a toolkit for FGFR signaling required for bud detachment.

## Discussion

### The evolutionary history of FGFR, Dof and Frs2

The receptor tyrosine kinase superfamily, to which the FGFRs belong, clearly predates multicellularity, as extensive families of RTKs are present in the unicellular holozoans *Capsaspora* and *Ministeria* (Suga et al. 2013) as well as in choanoflagellates such as *Monosiga* (Fairclough et al. 2013; King et al. 2008; Pincus et al. 2008). However, the RTKs of both *Capsaspora* and *Monosiga* have diverged independently from those of metazoans. The animal RTK types that respond to growth factors, including the fibroblast growth factor receptors, likely emerged in the eumetazoan ancestor (Bertrand et al. 2014; Rebscher et al. 2009).

**Fig. 4:**
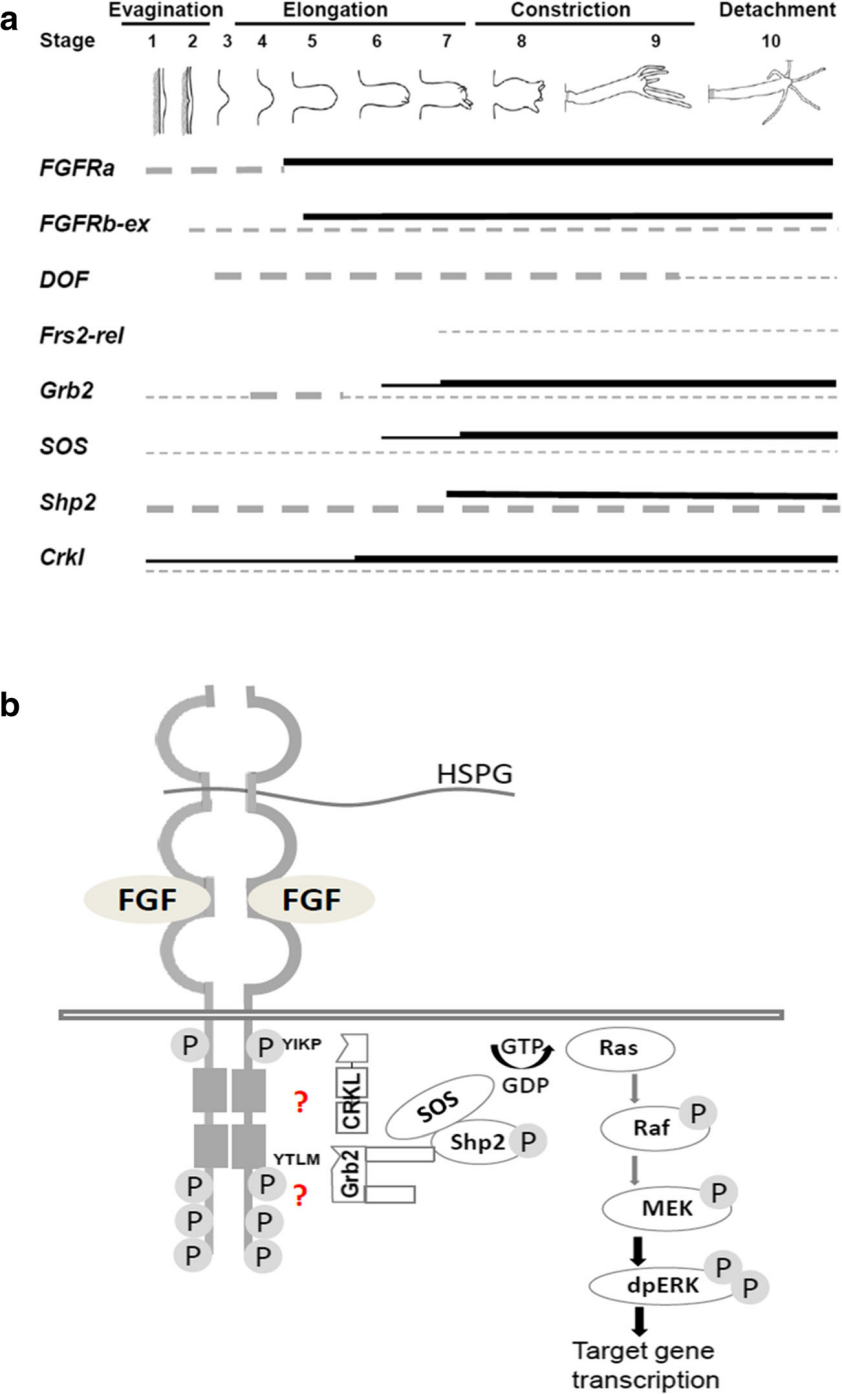
Schematic summary of the expression profile and of signaling pathways reached via potential *Hydra* FGFR downstream elements. (A) Time course of gene expression at the bud base ectoderm (continuous line) or elsewhere in the animal (dotted line). Expression intensity is indicated by line thickness. (B) Hypothetical alternative FGFR pathways in *Hydra* converging on the Ras/MAPK pathway. The SH2 domain-binding consensus sequences (YIKP, YTLM) in *Hydra* FGFR for direct binding of Grb2 or Crkl, respectively, are indicated

Animal RTKs generally require docking or adapter proteins to link an activated receptor dimer to intracellular pathways. This dependency is well established in *Drosophila* and vertebrates, where the FGFR docking proteins Dof or Frs2 are essential respectively, despite being unrelated in sequence. The apparent absence of both Dof and Frs2 from single-celled holozoans and choanoflagellates, as well as representatives of the Porifera, Ctenophora, and Placozoa, is consistent with the hypothesis of a eumetazoan origin of FGFRs (Bertrand et al. 2014). Clear homologues of Dof were identified in Cnidaria (*Hydra* and *Acropora*) and Bilateria, while Frs2 proteins sensu *strictu* were restricted to members of the Bilateria. Although Dof is not upregulated in the strong expression domains of the FGFRs in *Hydra*, *Crkl* expression is upregulated at the bud base near simultaneously with both of the FGFRs (Fig. 3; Fig. 4A), while upregulation of the adapter Grb2 as well as Sos and Shp2 occurs slightly later. This raises the possibility that two (Dof-independent) pathways operate at the bud base–the early phase involving Crkl and a later pathway in which Grb2 and Sos participate.

### Scenarios for FGFR adapter evolution

The data presented here suggest at least two possible scenarios for FGF adaptor evolution. In the first of these, the FGFR was originally coupled to downstream signaling pathways via an ancestral Dof protein*.* The evolutionary origin of Dof coincides with that of FGFs and FGFR sensu *strictu* in anthozoan and hydrozoan Cnidaria (Hasse et al. 2014; Matus et al. 2007; Rentzsch et al. 2008; Sudhop et al. 2004). As well as their overall similarity to known Dofs from Bilateria, the cnidarian Dof proteins contain all of the domains required for both docking to the activated FGFR and connecting with the downstream elements Grb2 and Shp2/Csw, all of which are consistent with the “Dof first” hypothesis. Mutant rescue experiments also provide some support for this idea. The *Hydra* FGFRa, partially rescued a fly *heartless* mutant (Rudolf et al. 2013), suggesting that the *Hydra* FGF receptor was capable of interacting with the *Drosophila* Dof adapter. However, the *Hydra* FGFR only rescued the very early phase of FGFR activity in fly embryos, prior to its involvement in MAPK signaling, which is absolutely dependent on Dof (Wilson et al. 2004). The fact that Dof shares a zone of expression with FGFRa (Kringelchen) only early in *Hydra* bud development raises the question of its function.

While it is possible that sufficient Dof protein is present to fulfill the FGFR docking function during bud detachment, a second scenario, in which *Hydra* FGFR requires neither Dof nor Frs2 for signal transduction, would account for the lack of Dof upregulation (Fig. 4B). This interesting alternative is supported by the fact that Grb2 acts as a direct interaction partner for several receptor tyrosine kinases including vertebrate FGFR2.

Grb2 is, for example, recruited directly to receptor tyrosine kinases like EGFR (Rozakis-Adcock et al. 1993). Moreover, the nematode *C. elegans* provides a precedent for FGFR signaling without Dof or Frs2. Neither is required for FGFR activity (and Dof not even encoded in the genome). Instead, the *C. elegans* FGFR Egl15 interacts directly with Grb2/SEM5 (Lo et al. 2010). Whether this mode of action reflects a secondary modification of the FGFR pathway is unclear. Interesting in this context is that Platyhelminthes also lack Dof and possess only atypical short Frs2-related proteins. How their FGFRs transduce signals into the cell would be interesting to know.

Last but not least, the unphosphorylated Grb2 tethers inactive FGFR2 dimers C-terminally and keeps them in a preactivation state (Belov and Mohammadi 2012; Lin et al. 2012). As soon as FGF ligands are available, the tyrosine phosphorylation of Grb2 induces its dissociation from the receptor and binding to Frs2 and only now, Grb2 acts as an adaptor for FGFR downstream signaling.

Both *Grb2* and *Crkl* have early origins, as clearly related proteins are encoded by sponge genomes. A protein similar to Grb2 is encoded by the *Salpingoeca* genome; thus, Grb2 might have functioned as an adapter for RTKs prior to the emergence of FGFRs. The *Hydra* FGFRs, Grb2 and Crkl, are all strongly transcribed in a ring of epithelial cells surrounding the late bud base that had been defined by Notch signaling (Münder et al. 2010), but Dof is not upregulated here. It will be interesting to test whether the second scenario, a direct interaction of FGFR with Grb2 (or Crkl) applies to FGFR signaling in Cnidaria and represents an ancestral mechanism.

## Conclusion

Efficient coupling of transmembrane receptors to intracellular signaling pathways often requires docking proteins, and many receptors are capable of interacting with a number of different downstream adaptors. The ability to interact with multiple downstream proteins enables receptors to regulate a range of different functions; in the case of FGFRs, these include roles in cell proliferation, differentiation, migration, boundary formation, and branching morphogenesis. Although the evolutionary origins of the Dof protein coincided with those of FGF signaling, and the cnidarian Dof has all of the structural features required to function as an FGFR docking protein, it missing upregulation at the bud base raises the possibility that it does not fulfill that role in *Hydra*. Protein–protein interaction assays are now required to identify and functionally characterize the interaction partners of *Hydra* FGFR required for signal transduction.

## Electronic supplementary material

ESM 1 AB: Summary of FRS2 and Dof proteins. (A) Accession numbers of FRS2 and Dof proteins. (B) Schematic summary of the domain structure of predicted Frs2related proteins in invertebrates. These proteins clearly belong to the membrane-linked proteins (MBP). (PNG 184 kb)

High Resolution Image (TIF 2210 kb)

ESM 1 B (PNG 193 kb)

High Resolution Image (TIF 931 kb)

ESM 2 ABC: Domain structure and alignment of the PTB domain of membranelinked proteins (MLP). (A) Schematic domain structure of MLPs including Dok, FRS2, IRS and FRS2-related proteins. (B) Alignment of the PTB domain of MLPs. Indicated are the insulin receptor binding residues as defined for vertebrate IRS proteins in comparison to Dok, Frs2 and Frs2-related members of the MLP superfamily. (C) Names and database accession numbers of membrane-linked Dok and IRS proteins. (PNG 83 kb)

High Resolution Image (TIF 522 kb)

ESM 2 C (PNG 74 kb)

High Resolution Image (TIFF 266 kb)

ESM 3 Alignment of the DBB motif and ankyrin repeats of Dof proteins. (PNG 838 kb)

High Resolution Image (TIF 15494 kb)

ESM 4: Cnidarian DOF sequences and their predicted SH2, SH3 binding site consensus sequences. (PNG 465 kb)

High Resolution Image (TIF 1039 kb)

ESM 5 Name and database accession numbers of the used Grb2, Shp2/Csw (Pfam 00102), Sos and Crkl sequences. Asterisks indicate sequences included for the domain summary in Fig. S6 (PNG 79 kb)

High Resolution Image (TIF 378 kb)

ESM 6 ABCD: Schematic summary of signaling elements downstream of FGFR. (A) Schematic representation of alternative pathway elements downstream of vertebrate or fly FGFR targeting Ras/MAPK signaling. (B-D) Domain structure of the tyrosine phosphatase Shp2/Csw (B), the adapter Grb2 (C) and the dual function Rac/Rho GEF, Sos (D). (PNG 242 kb)

High Resolution Image (TIF 1447 kb)

ESM 7 AB: Schematic summary of CRK and CRKL adapter proteins for FGFR, their domains and phylogenetic tree. (A) Schematic representation of Crk and Crkl domains with their SH2 (red), SH3 (blue) binding domains and the tyrosine residue (yellow) necessary for activation of Crkl binding (Feller et al. 1994). (B) Phylogenetic tree of Crk and Crkl proteins. Aq Amphimedon queenslandica, Bf Branchiostoma floridae, Ce Caenorhabditis elegans, Cg Crassostrea gigas, Ci Ciona intestinalis, Dm Drosophila melanogaster, Dr Danio rerio, Hs: Homo sapiens, Hv Hydra vulgaris, Mm Mus musculus, Nv Nematostella vectensis, Sk Saccoglossus kowalevskii, Xl Xenopus laevis. (PNG 145 kb)

High Resolution Image (TIF 800 kb)

ESM 8 Sense- and antisense controls and examples for staining artefacts during in situ hybridization to detect the gene expression patterns of potential FGFR downstream elements. (A, K) Hym176 (Yum et al. 1997) antisense control (neurons), (B) Hym176 sense control; (C – H) Sense controls for Crkl, Grb2, Dof, Frs2, Shp2 and Sos. The open arrowhead additionally indicates examples of artefactual staining observed sometimes with sense and antisense probes in ~10% of the animals: (E, H) broken tissue binds probe or antibody, (F) extracellular signal in a mucus-like structure attached to the basal disc, (H) probe or antibody trapping in broken endoderm. (I, J) Whole mount pattern of Grb2 transcripts (I = Fig.3E1) in budding polyps: Above evaginating young buds an initially circumferential zone (I) of strong expression establishes. (K) Close-up of (A), neurons above the basal disc are stained blue. Color development was allowed for 5 min (A, K) or 2:20 hrs (B-J). (PNG 715 kb)

High Resolution Image (TIF 3047 kb)
