# Supplementary figures and images for: What lies beneath: Hydra provides cnidarian perspectives into the evolution of FGFR docking proteins
Source: Dev Genes Evol. 2020 Mar 20;230(3):227–38. doi: 10.1007/s00427-020-00659-4 (PMC7260276; doi:10.1007/s00427-020-00659-4)

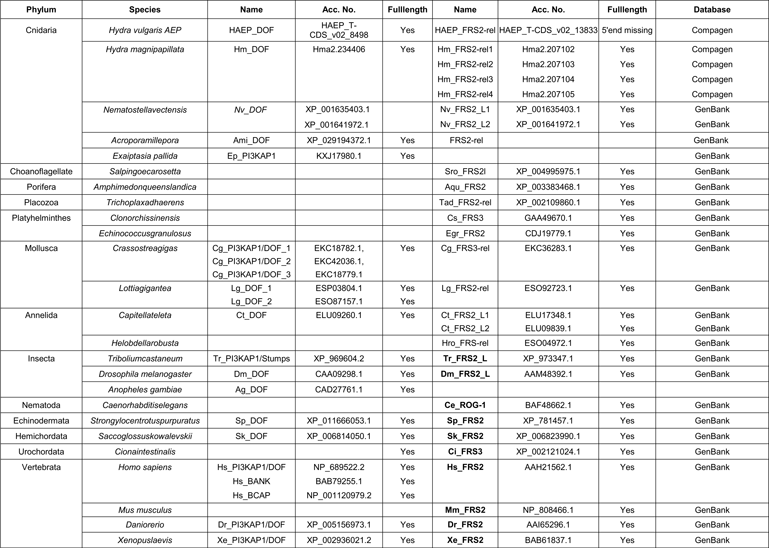

Supplement: Supplementary file 1 — AB: Summary of FRS2 and Dof proteins. (A) Accession numbers of FRS2 and Dof proteins. (B) Schematic summary of the domain structure of predicted Frs2related proteins in invertebrates. These proteins clearly belong to the membrane-linked proteins (MBP). (PNG 184 kb) [file 427_2020_659_Fig5_ESM.png]

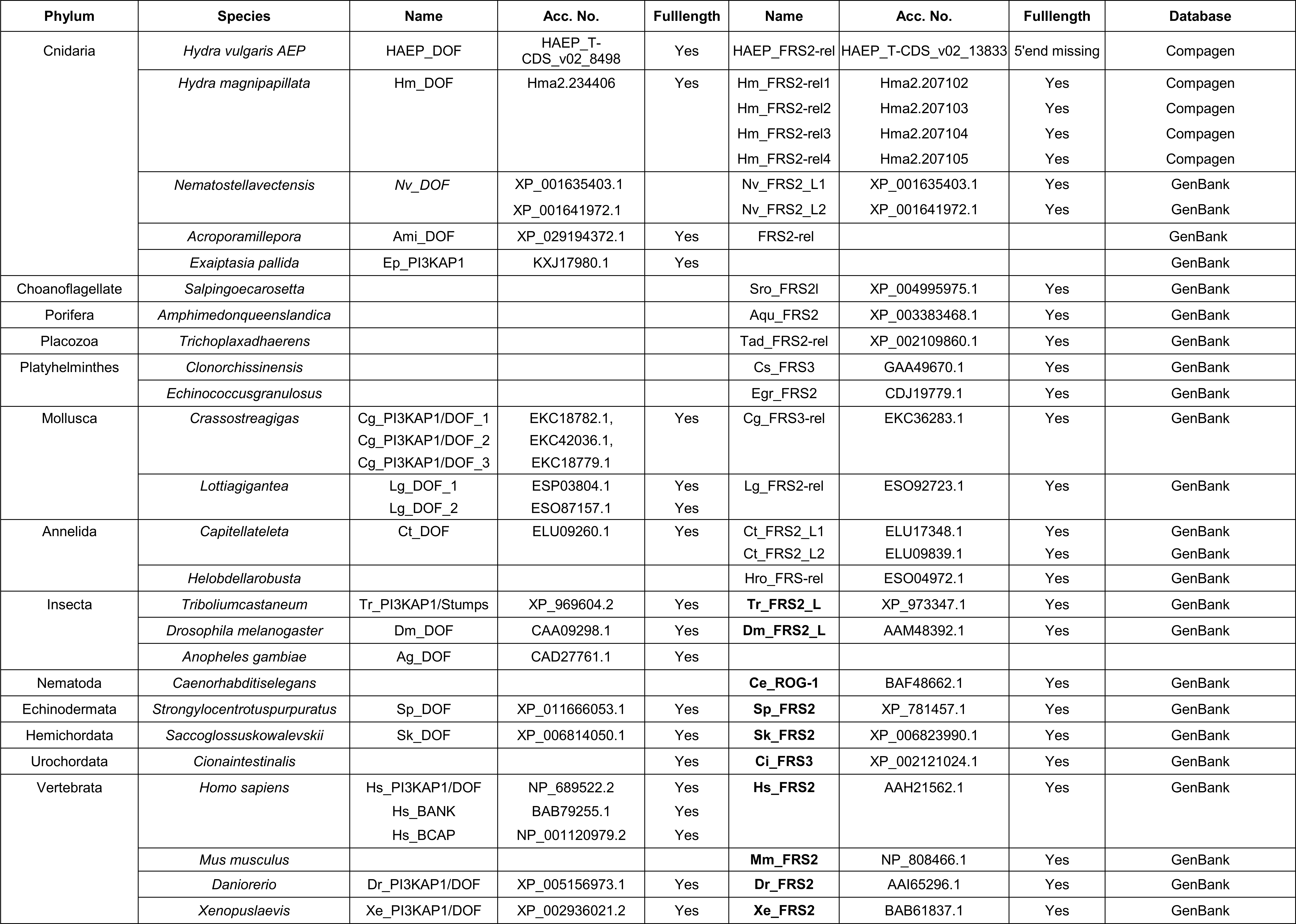

Supplement: Supplementary file 2 — High Resolution Image (TIF 2210 kb) [file 427_2020_659_MOESM1_ESM.tif]

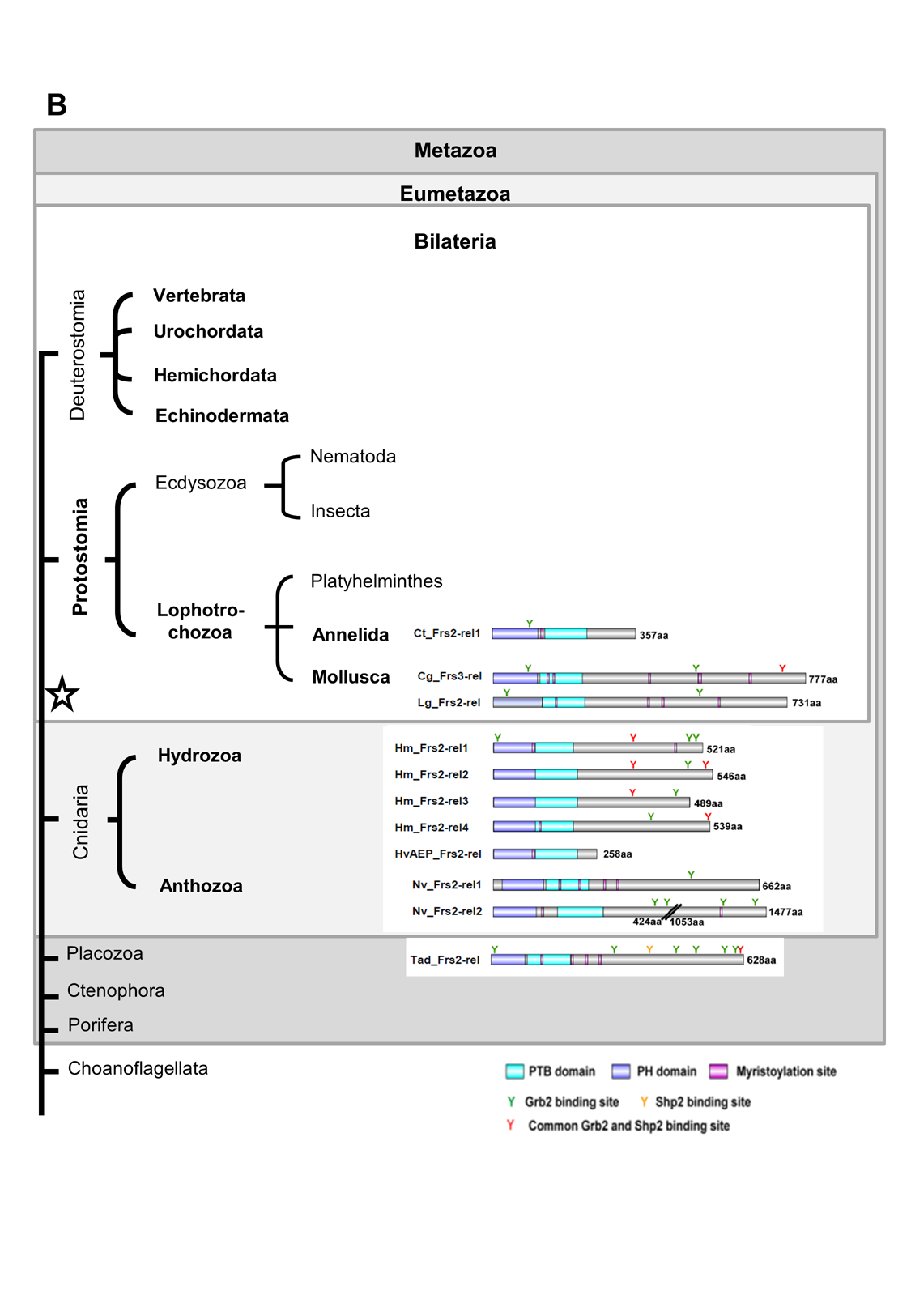

Supplement: Supplementary file 3 — ESM 1 B (PNG 193 kb) [file 427_2020_659_Fig6_ESM.png]

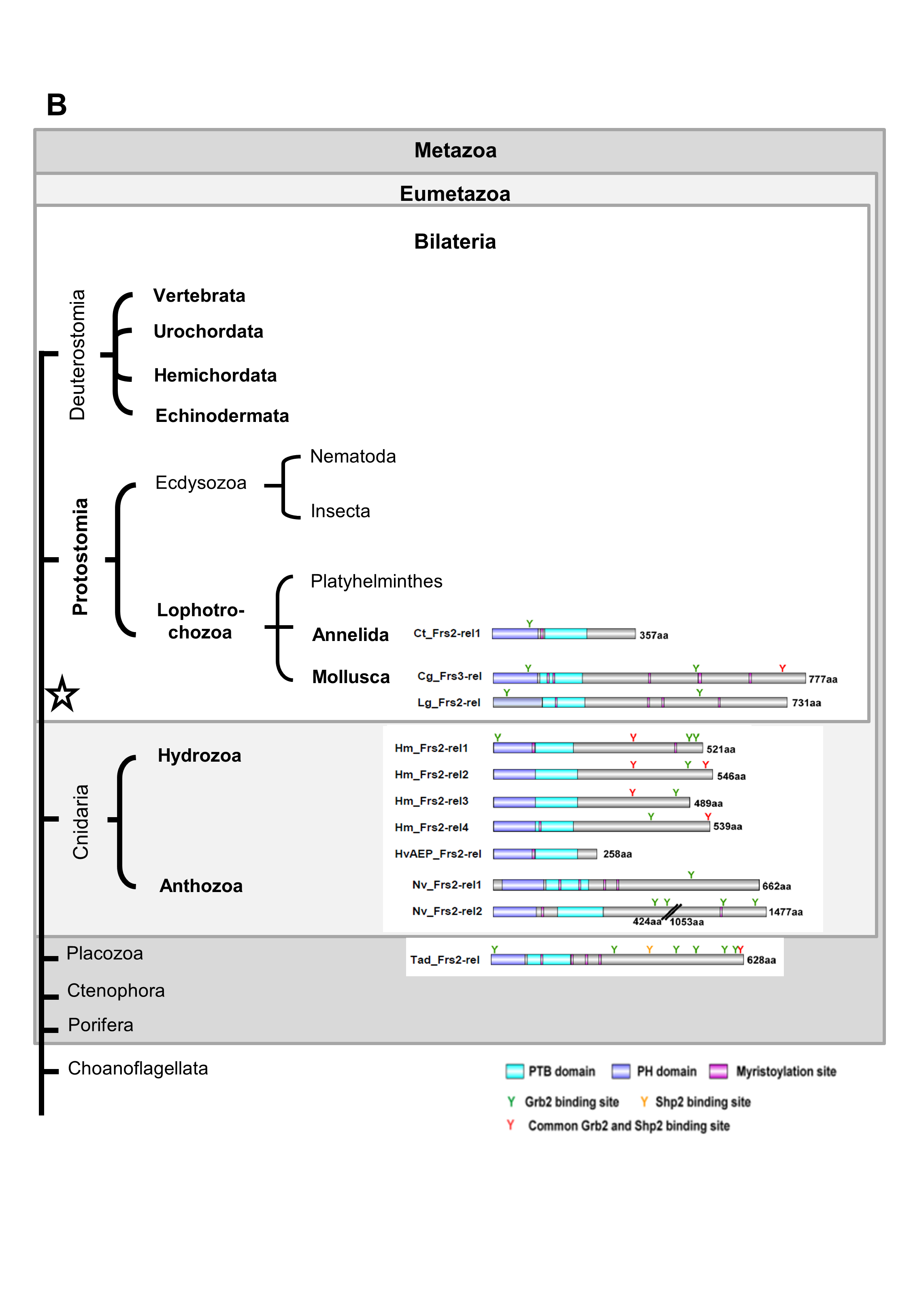

Supplement: Supplementary file 4 — High Resolution Image (TIF 931 kb) [file 427_2020_659_MOESM2_ESM.tif]

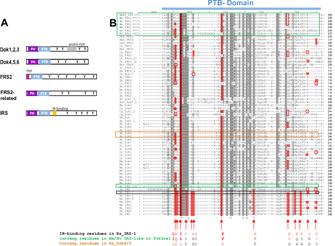

Supplement: Supplementary file 5 — ABC: Domain structure and alignment of the PTB domain of membranelinked proteins (MLP). (A) Schematic domain structure of MLPs including Dok, FRS2, IRS and FRS2-related proteins. (B) Alignment of the PTB domain of MLPs. Indicated are the insulin receptor binding residues as defined for vertebrate IRS proteins in comparison to Dok, Frs2 and Frs2-related members of the MLP superfamily. (C) Names and database accession numbers of membrane-linked Dok and IRS proteins. (PNG 83 kb) [file 427_2020_659_Fig7_ESM.png]

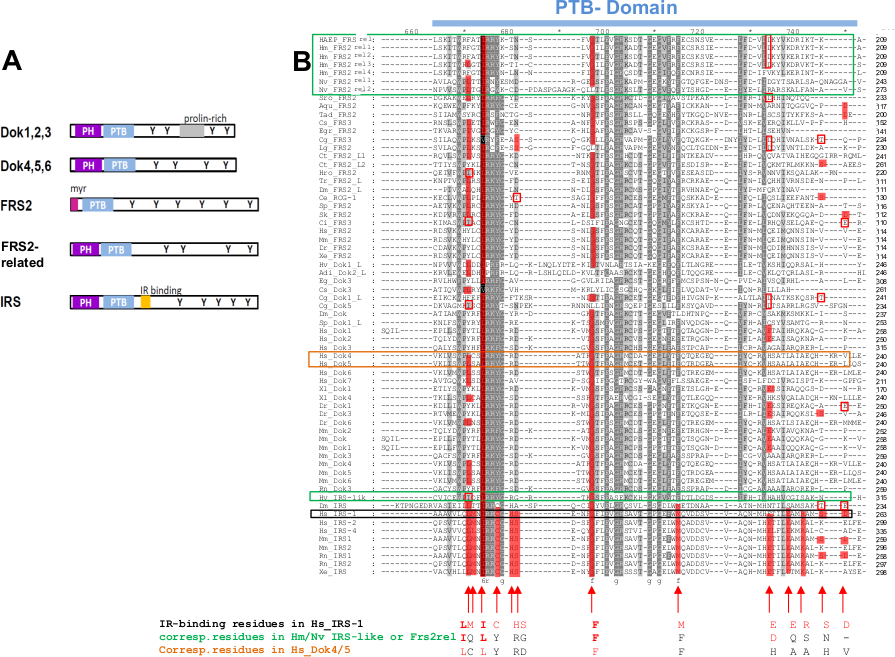

Supplement: Supplementary file 6 — High Resolution Image (TIF 522 kb) [file 427_2020_659_MOESM3_ESM.tif]

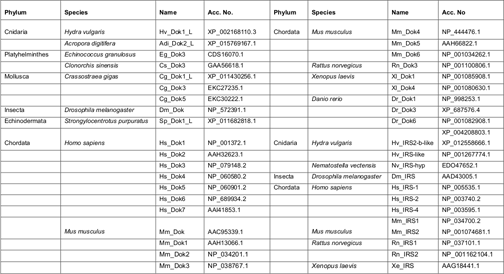

Supplement: Supplementary file 7 — ESM 2 C (PNG 74 kb) [file 427_2020_659_Fig8_ESM.png]

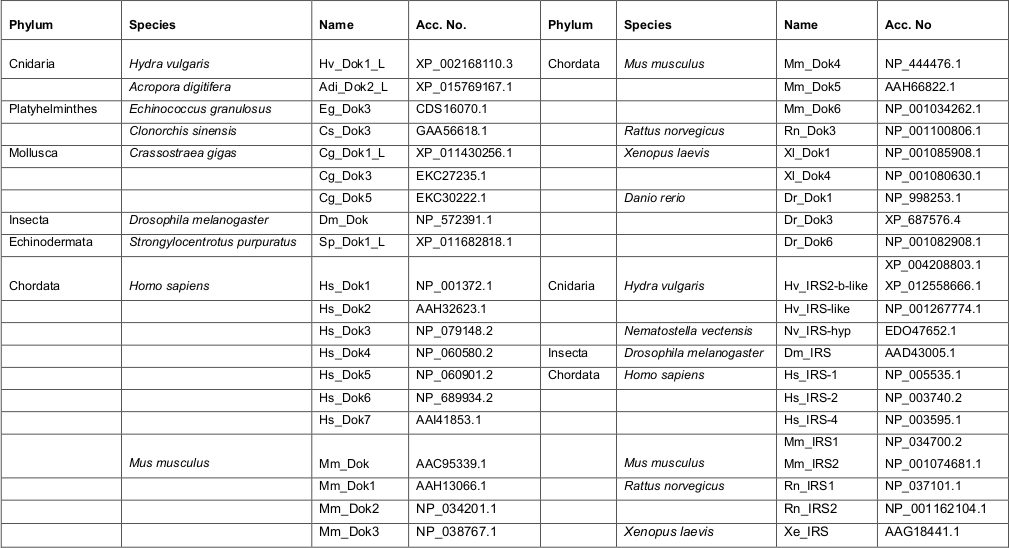

Supplement: Supplementary file 8 — High Resolution Image (TIFF 266 kb) [file 427_2020_659_MOESM4_ESM.tiff]

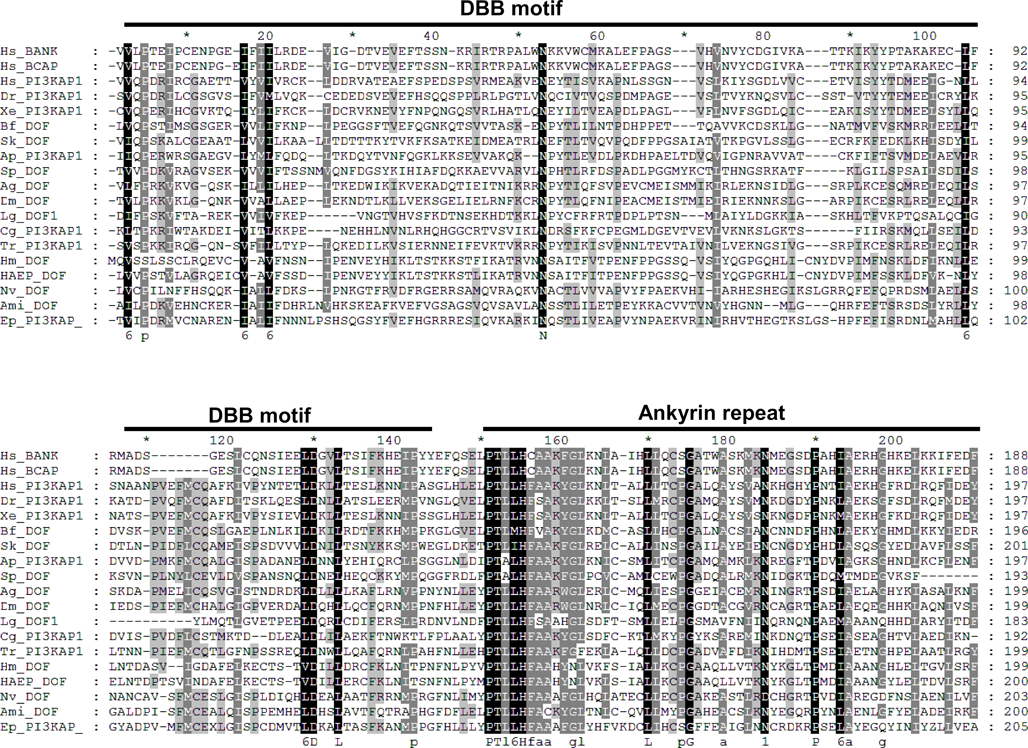

Supplement: Supplementary file 9 — Alignment of the DBB motif and ankyrin repeats of Dof proteins. (PNG 838 kb) [file 427_2020_659_Fig9_ESM.png]

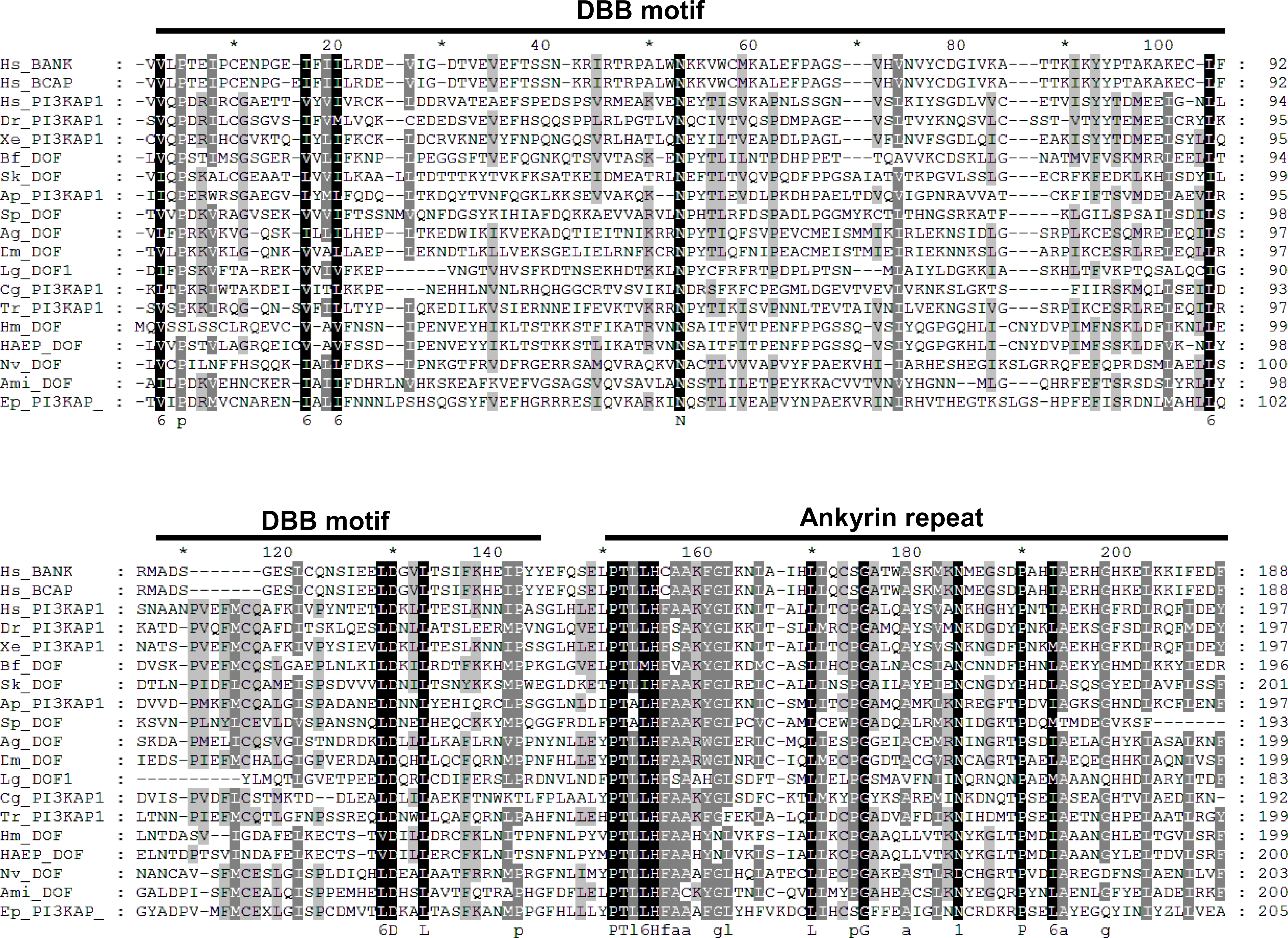

Supplement: Supplementary file 10 — High Resolution Image (TIF 15494 kb) [file 427_2020_659_MOESM5_ESM.tif]

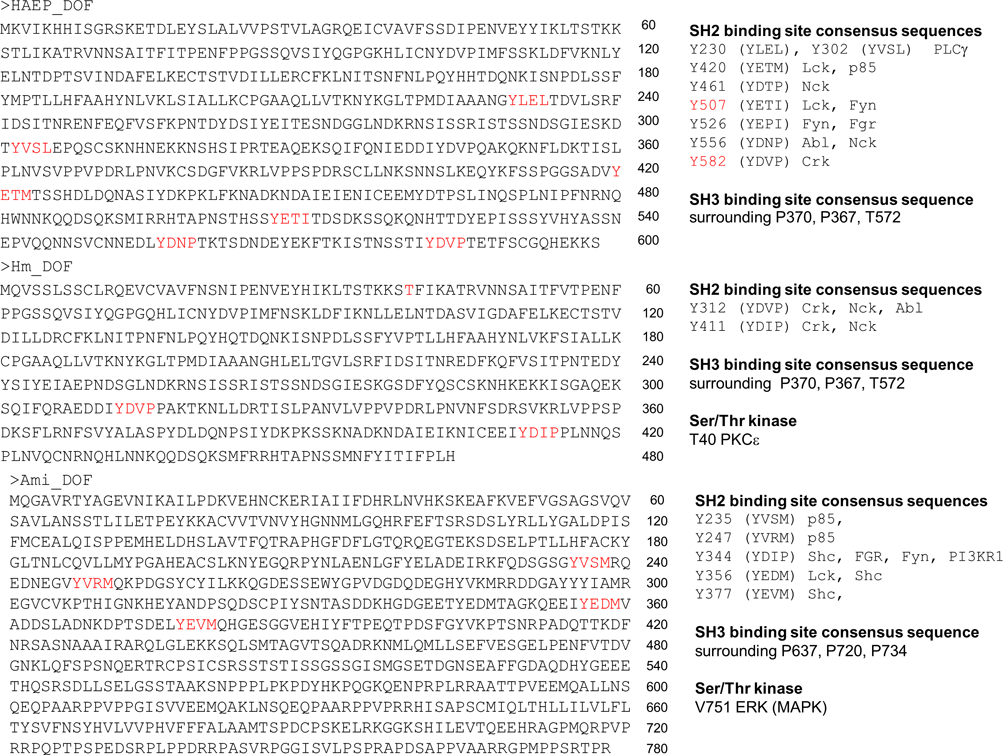

Supplement: Supplementary file 11 — Cnidarian DOF sequences and their predicted SH2, SH3 binding site consensus sequences. (PNG 465 kb) [file 427_2020_659_Fig10_ESM.png]

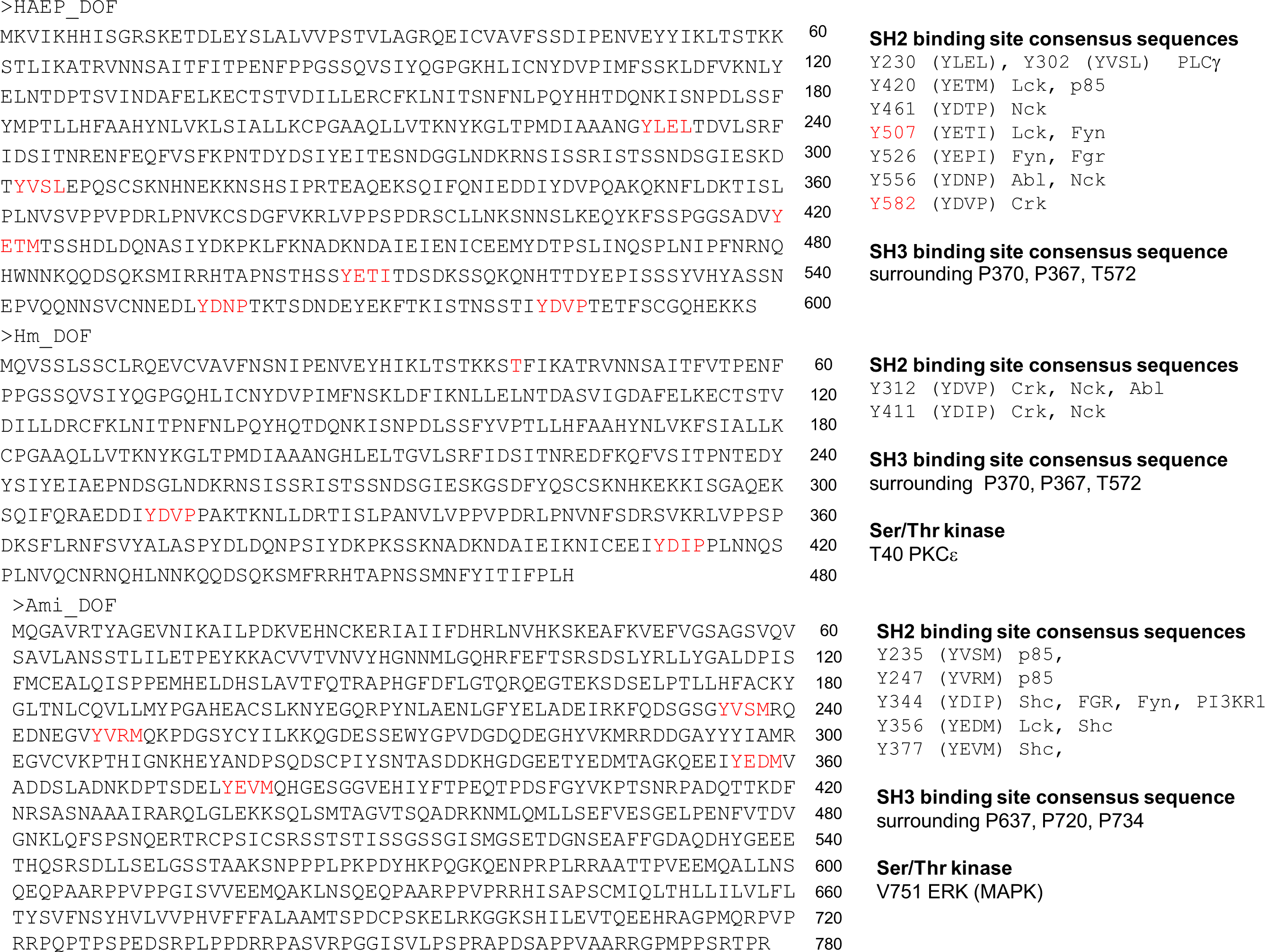

Supplement: Supplementary file 12 — High Resolution Image (TIF 1039 kb) [file 427_2020_659_MOESM6_ESM.tif]

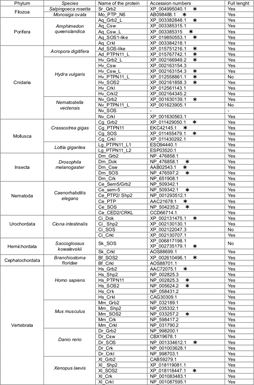

Supplement: Supplementary file 13 — Name and database accession numbers of the used Grb2, Shp2/Csw (Pfam 00102), Sos and Crkl sequences. Asterisks indicate sequences included for the domain summary in Fig. S6 (PNG 79 kb) [file 427_2020_659_Fig11_ESM.png]

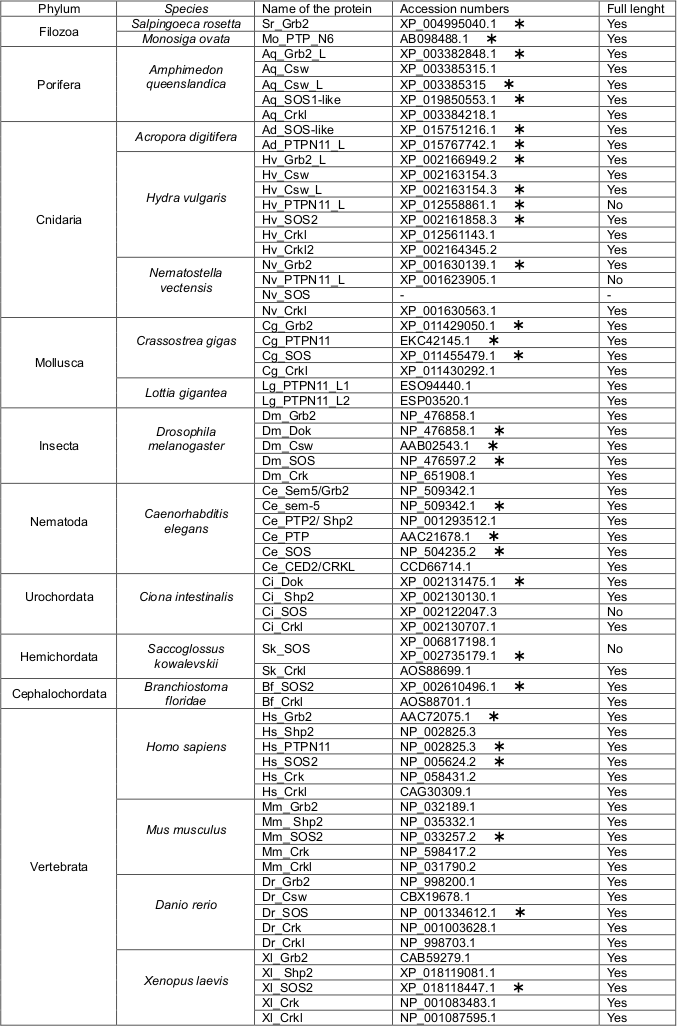

Supplement: Supplementary file 14 — High Resolution Image (TIF 378 kb) [file 427_2020_659_MOESM7_ESM.tif]

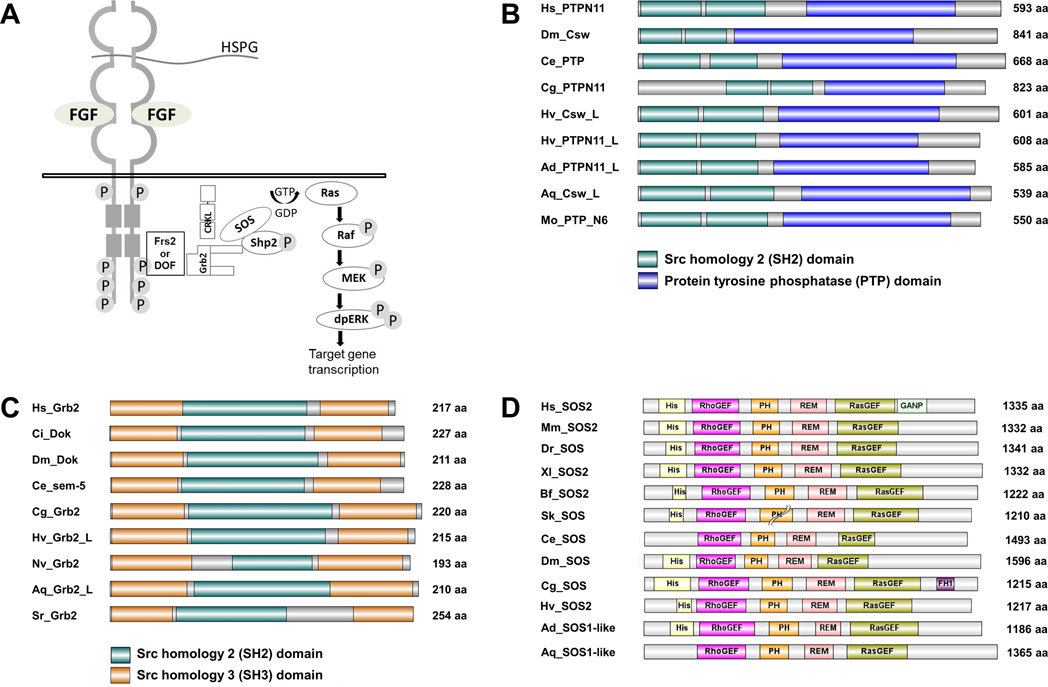

Supplement: Supplementary file 15 — ABCD: Schematic summary of signaling elements downstream of FGFR. (A) Schematic representation of alternative pathway elements downstream of vertebrate or fly FGFR targeting Ras/MAPK signaling. (B-D) Domain structure of the tyrosine phosphatase Shp2/Csw (B), the adapter Grb2 (C) and the dual function Rac/Rho GEF, Sos (D). (PNG 242 kb) [file 427_2020_659_Fig12_ESM.png]

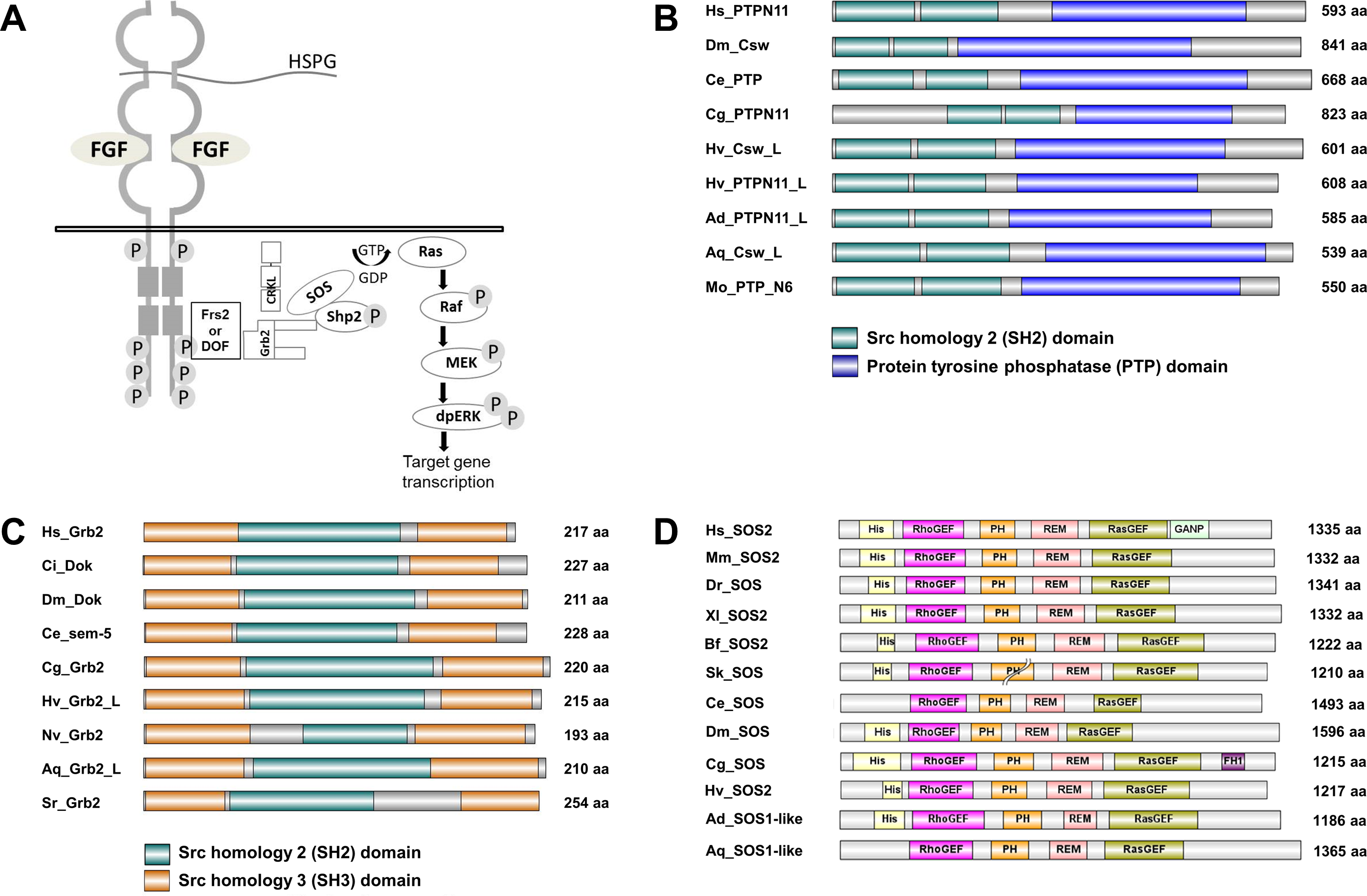

Supplement: Supplementary file 16 — High Resolution Image (TIF 1447 kb) [file 427_2020_659_MOESM8_ESM.tif]

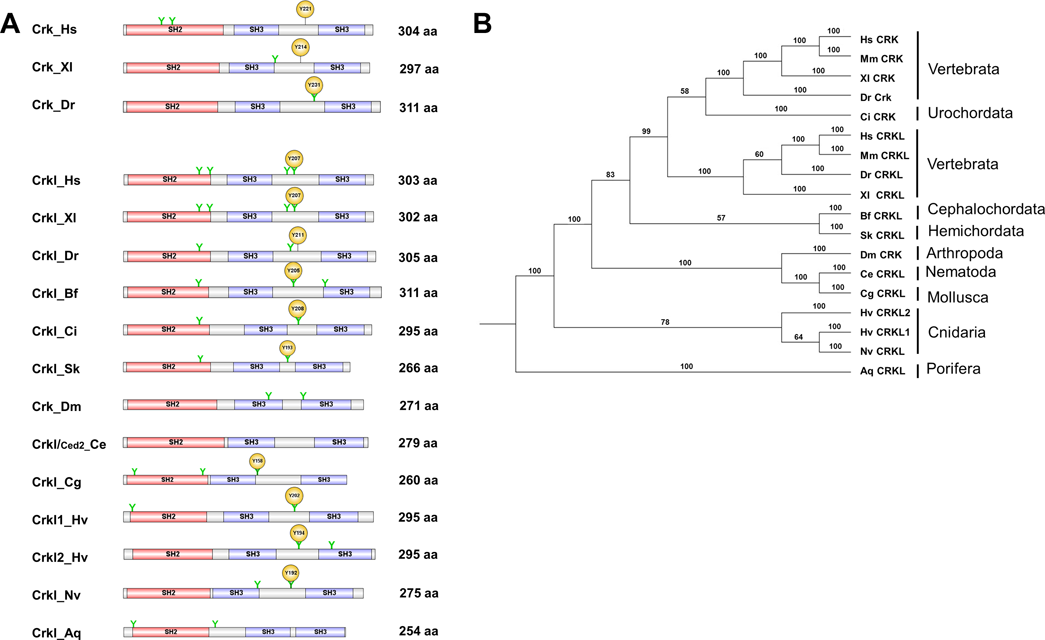

Supplement: Supplementary file 17 — AB: Schematic summary of CRK and CRKL adapter proteins for FGFR, their domains and phylogenetic tree. (A) Schematic representation of Crk and Crkl domains with their SH2 (red), SH3 (blue) binding domains and the tyrosine residue (yellow) necessary for activation of Crkl binding (Feller et al. 1994). (B) Phylogenetic tree of Crk and Crkl proteins. Aq Amphimedon queenslandica, Bf Branchiostoma floridae, Ce Caenorhabditis elegans, Cg Crassostrea gigas, Ci Ciona intestinalis, Dm Drosophila melanogaster, Dr Danio rerio, Hs: Homo sapiens, Hv Hydra vulgaris, Mm Mus musculus, Nv Nematostella vectensis, Sk Saccoglossus kowalevskii, Xl Xenopus laevis. (PNG 145 kb) [file 427_2020_659_Fig13_ESM.png]

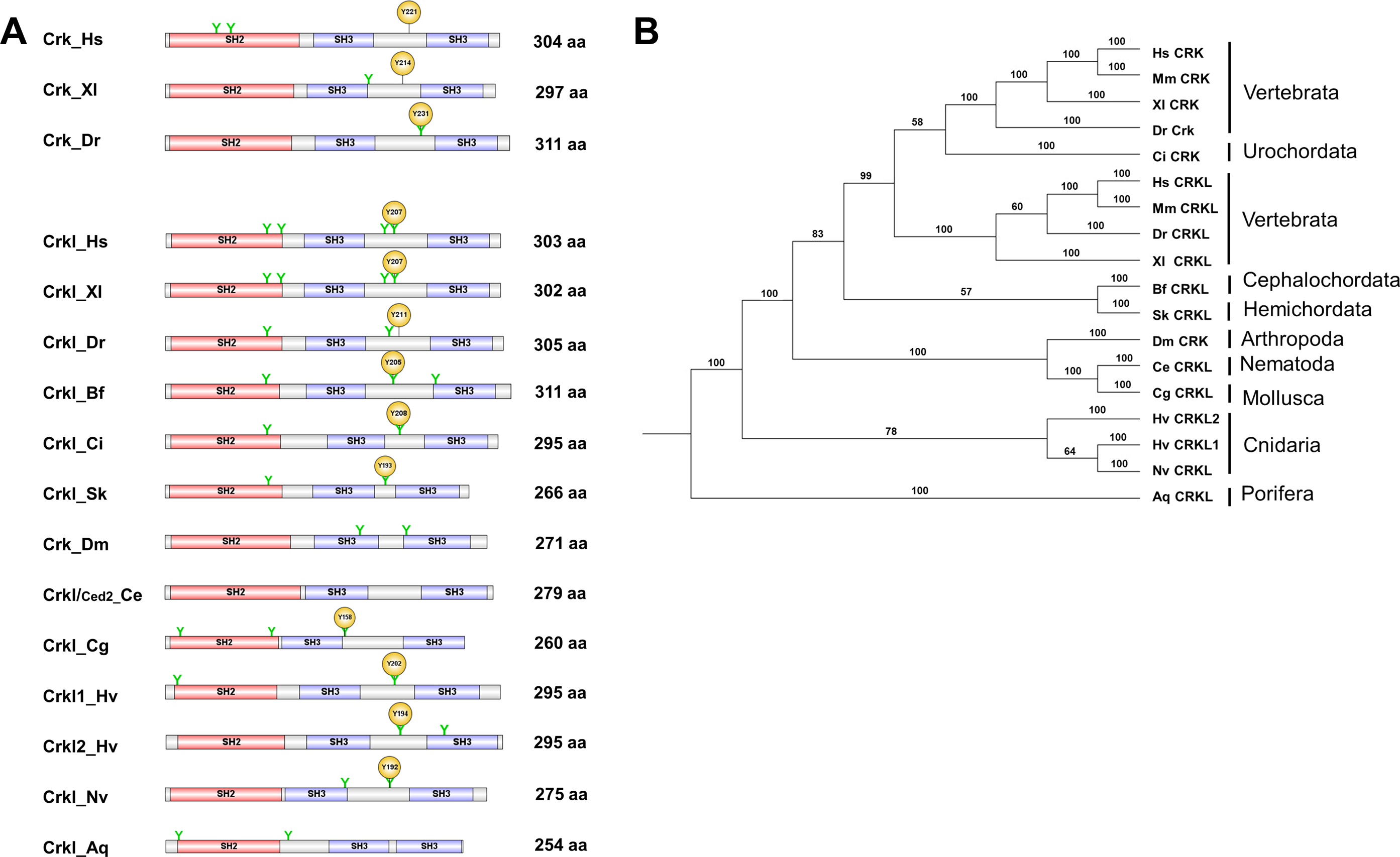

Supplement: Supplementary file 18 — High Resolution Image (TIF 800 kb) [file 427_2020_659_MOESM9_ESM.tif]

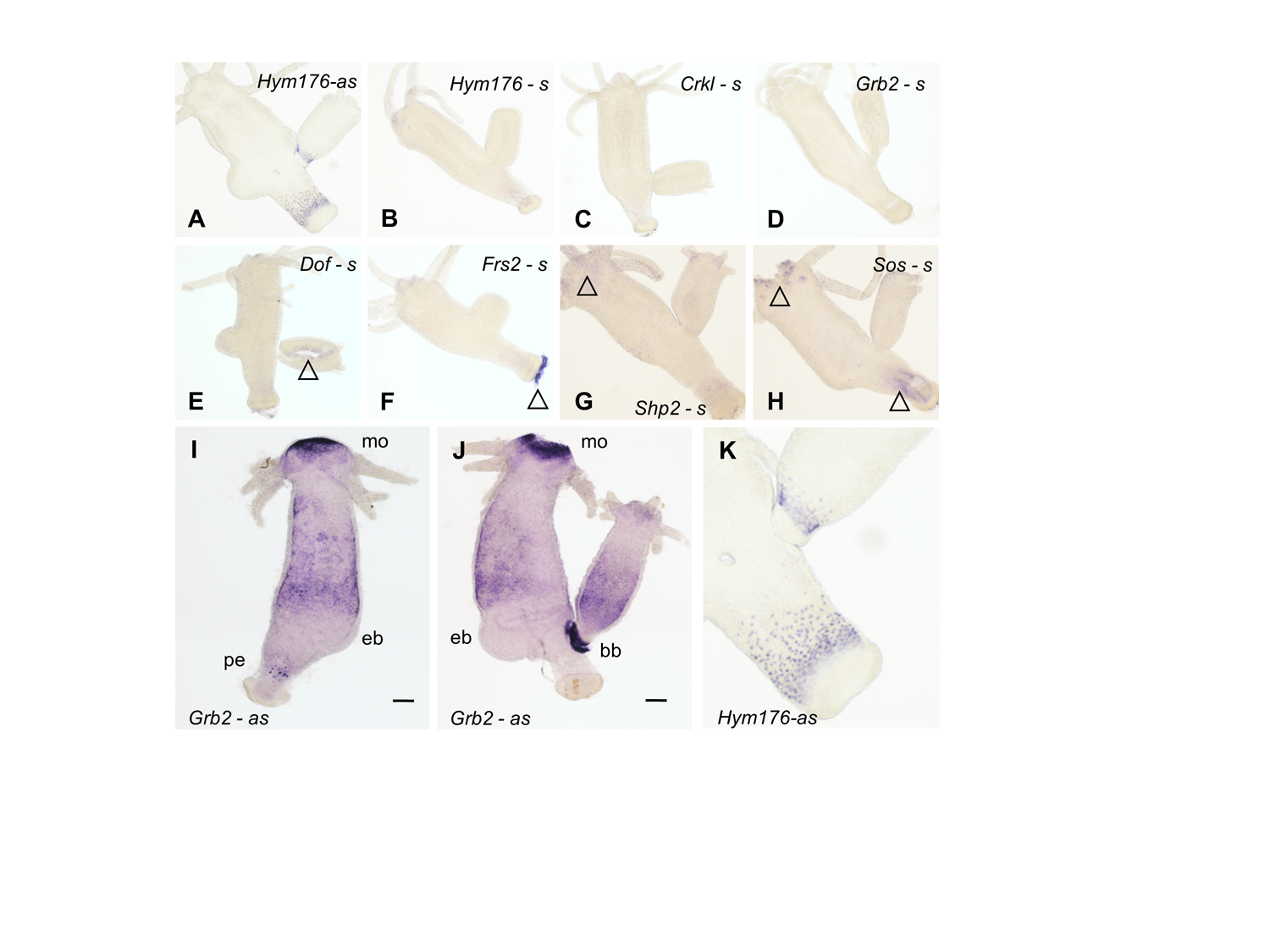

Supplement: Supplementary file 19 — Sense- and antisense controls and examples for staining artefacts during in situ hybridization to detect the gene expression patterns of potential FGFR downstream elements. (A, K) Hym176 (Yum et al. 1997) antisense control (neurons), (B) Hym176 sense control; (C – H) Sense controls for Crkl, Grb2, Dof, Frs2, Shp2 and Sos. The open arrowhead additionally indicates examples of artefactual staining observed sometimes with sense and antisense probes in ~10% of the animals: (E, H) broken tissue binds probe or antibody, (F) extracellular signal in a mucus-like structure attached to the basal disc, (H) probe or antibody trapping in broken endoderm. (I, J) Whole mount pattern of Grb2 transcripts (I = Fig.3E1) in budding polyps: Above evaginating young buds an initially circumferential zone (I) of strong expression establishes. (K) Close-up of (A), neurons above the basal disc are stained blue. Color development was allowed for 5 min (A, K) or 2:20 hrs (B-J). (PNG 715 kb) [file 427_2020_659_Fig14_ESM.png]

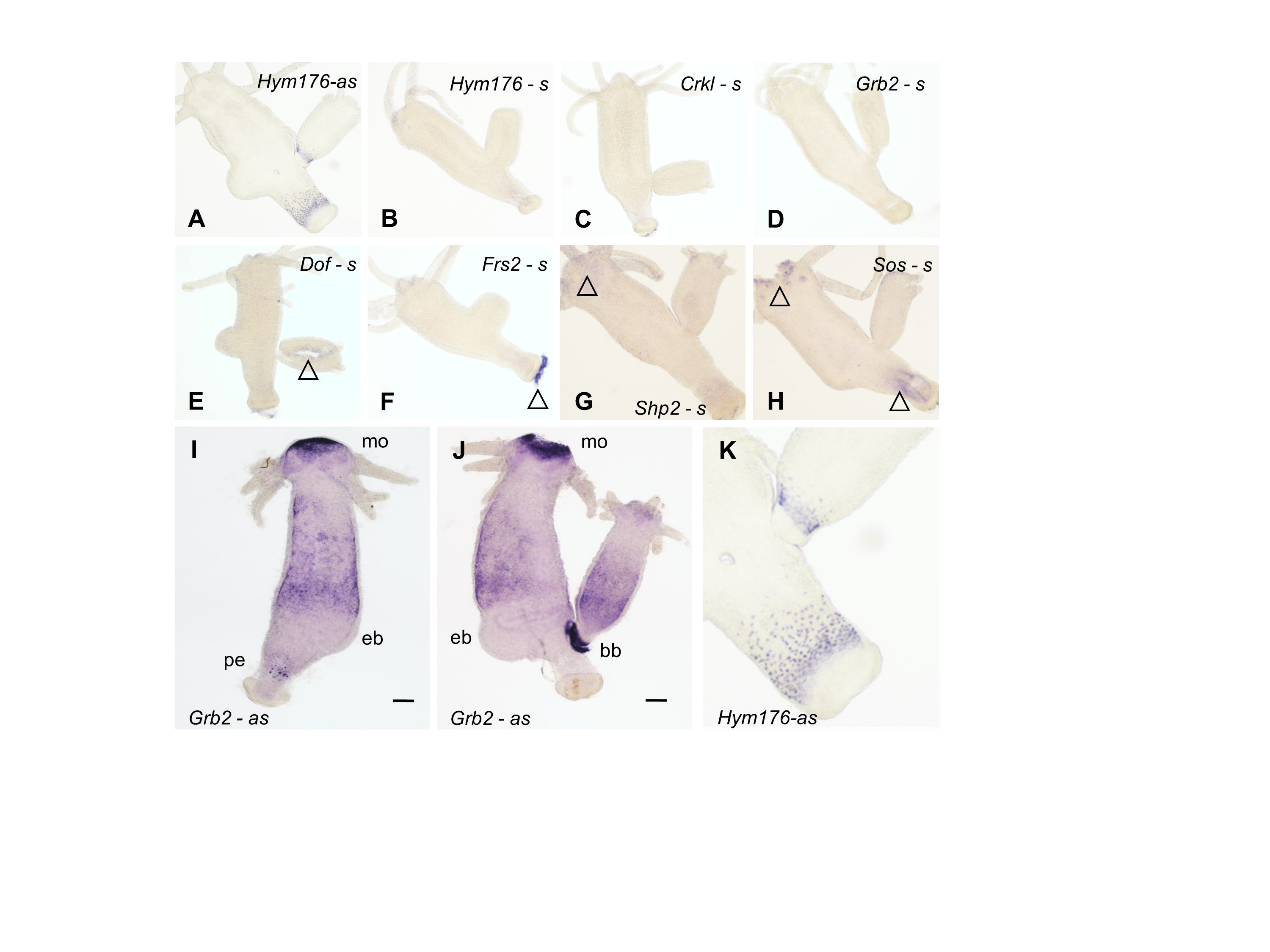

Supplement: Supplementary file 20 — High Resolution Image (TIF 3047 kb) [file 427_2020_659_MOESM10_ESM.tif]
